# Direct imaging Berry curvature–induced transport and domain behavior in noncollinear single-crystal antiferromagnetic thin films

**DOI:** 10.1126/sciadv.aeb6478

**Published:** 2026-08-26

**Authors:** Yuchuan Yao, Pratap Pal, Camron Farhang, Weihang Lu, Mohamed Elekhtiar, Paul Lenharth, Neil G. Campbell, Gautam Gurung, Roger D. Johnson, Pascal Manuel, Mark S. Rzchowski, Evgeny Y. Tsymbal, Jing Xia, Chang-Beom Eom

**Affiliations:** ^1^ Department of Materials Science and Engineering, University of Wisconsin-Madison, WI 53706, USA.; ^2^ Department of Physics and Astronomy, University of California, Irvine, CA 92696, USA.; ^3^ Department of Physics and Astronomy and Nebraska Center for Materials and Nanoscience, University of Nebraska, Lincoln, NE 68588, USA.; ^4^ Department of Physics, University of Wisconsin-Madison, WI 53706, USA.; ^5^ Clarendon Laboratory, Department of Physics, University of Oxford, Parks Road, Oxford OX1 3PU, UK.; ^6^ Trinity College, University of Oxford, Oxford OX1 3BH, UK.; ^7^ Department of Physics and Astronomy, University College London, London, WC1E 6BT, UK.; ^8^ London Centre for Nanotechnology, University College London, London WC1E 6BT, UK.; ^9^ ISIS Facility, STFC Rutherford Appleton Laboratory, Didcot, Oxfordshire OX11 0QX, UK.

## Abstract

The discovery of the intrinsic anomalous Hall effect (AHE) in noncollinear antiferromagnets has opened a plethora of promising opportunities in antiferromagnetic devices. The key challenges limiting their full potential are (i) high-quality epitaxial thin-film growth and (ii) the understanding of Berry curvature and antiferromagnetic domain physics. Here, we focus on a noncollinear antiperovskite antiferromagnet Mn_3_NiN as a model system, successfully grown as a single-crystal epitaxial thin film. Combining multiple experiments supported by theoretical calculations, we probe the Berry curvature associated with antiferromagnetic Γ_4g_ domains in Mn_3_NiN and its strong connection to an AHE. We directly image the antiferromagnetic domains driving the intrinsic Berry curvature with high-resolution Sagnac MOKE (magneto-optical Kerr-effect) microscopy, controlling spatial distribution and dynamics by varying temperature and applied magnetic fields. Our findings provide critical advancement of the fundamental understanding and wide tunability of Berry curvature in noncollinear antiferromagnets important for realization in potential spintronic applications.

## INTRODUCTION

Understanding and manipulating the coupling of electronic spin and charge in antiferromagnets offers enormous potential applications with the advantages of lack of stray fields, low susceptibility to external perturbations, and ultrafast spin dynamics (*1*–*10*). However, the absence of net magnetization presents substantial challenges for the detection and control of the antiferromagnetic state (*3*). Recent progress has come from the discovery of a large anomalous Hall effect (AHE) in noncollinear antiferromagnets (NCAFs) such as Mn-based hexagonal Mn_3_Sn (*11*) or Mn_3_Ge (*12*), cubic Mn_3_Pt (*13*) or Mn_3_Ir, (*14*) and antiperovskites Mn_3_*X*N [*X* = Ga (*15*), Sn (*16*), and Ni (*17*)]. These NCAFs have a nearly compensated moment or a very weak spin-orbit coupling–driven tilted moment in or out of the (111) Kagome plane (*9*, *11*), thus precluding a simple origin of the AHE in these systems driven by the net magnetic moment. Recent theoretical studies have highlighted the role of spin-orbit coupling with the simultaneous absence of symmetries (e.g., mirror symmetries in Γ_4g_ phase of Mn_3_NiN) in making the sum of Berry curvature over the Brillouin zone nonzero (*18*, *19*), resulting in a nonzero AHE. Thus, it raises a question of what drives the AHE in such NCAFs and how to control it like conventional ferromagnetic materials for information writing.

Although the information writing/reading via controlling magnetic domains is well established in conventional ferromagnets (*20*), such control in an antiferromagnet is extremely challenging. However, some recent progress has shown promise for the noncollinear antiferromagnet (Mn_3_Sn), where magnetic domains were mapped via magneto-optical Kerr-effect (MOKE) imaging (*21*) in a bulk sample. However, the hexagonal structure and complex magnetic configuration of Mn_3_Sn not only complicates understanding but also brings substantial challenges for epitaxial thin-film growth (*11*). Use of NCAF materials with large AHE for spintronics requires epitaxial growth of these materials with simple crystal and spin structures. This not only provides better understanding but also offers simpler integration with other functional materials in a device heterostructure.

**Fig. 1.**
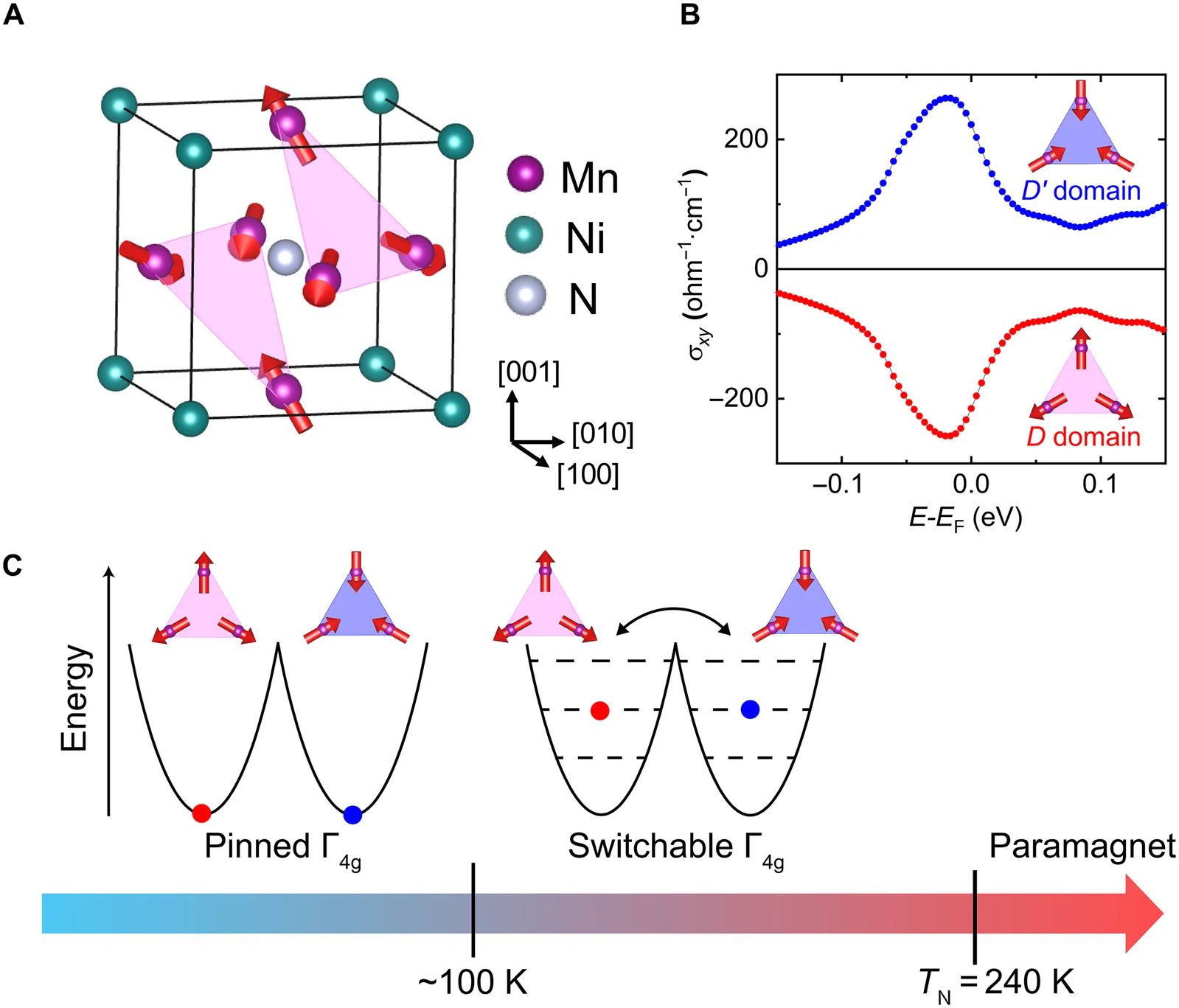
Schematic illustration of unusual temperature-dependent Γ_4g_ spin order in Mn_3_NiN. (**A**) Unit cell crystal structure of antiperovskite Mn_3_NiN with Γ_4g_ spin configuration. (**B**) Two different types of magnetic domains with time-reversal symmetry in Mn_3_NiN: *D* where spins point out and *D*′ where spins point in. Corresponding AHC for these two types of Γ_4g_ spin configurations in Mn_3_NiN, as deduced by DFT calculations. (**C**) Unusual temperature-dependent response in Mn_3_NiN thin films due to strong anisotropy of magnetic domains.

Here, we choose Mn_3_NiN with antiperovskite structure as a model system, which has a simple cubic crystal structure with lattice parameter close to commercially available substrates and Γ_4g_ triangular spin configuration (*11*, *22*). Such a spin configuration breaks time-reversal symmetry, resulting in a nonvanishing Berry curvature, as found in our first-principles density functional theory (DFT) calculations (fig. S1). Mn_3_NiN hosts eight possible magnetic domains (*10*, *23*–*25*), which can be sorted into two different kinds of magnetic domains (*D* and *D*′) due to time reversal symmetry (fig. S2). This gives rise to positive and negative AHE (Fig. 1B), originating from opposite chiralities of the spin configurations (*25*, *26*). Such binary response of AHE, despite the antiferromagnetic nature, makes Mn_3_NiN a very compelling model system for information writing in a simple “0” and “1” scheme (*27*). Moreover, Mn_3_NiN is an interesting and promising material platform not only for fundamental investigations into the nature of the Berry curvature and antiferromagnetic domains, but also for achieving efficient and deterministic magnetic domain writing. To overcome the limited spatial resolution of conventional MOKE, we use a zero-loop Sagnac interferometer with unprecedented MOKE sensitivity (*28*) to directly image smaller NCAF Γ_4g_ magnetic domains responsible for Berry phase AHE in Mn_3_NiN thin film. This provides direct evidence of unusual temperature-dependent AHE in Mn_3_NiN thin film. Combining our temperature-dependent transport measurements and theoretical calculations, we provide a comprehensive framework for spintronic applications.

We demonstrate the growth of high-quality atomically smooth epitaxial Mn_3_NiN thin films, establish their robust antiferromagnetic properties, and observe a large AHE, which is strongly correlated with the underlying nanoscale Berry curvature and antiferromagnetic domain texture. Our films show noncollinear Γ_4g_ magnetic ordering below the Néel temperature (*T*_N_) of 240 K that majorly retains this symmetry to the lowest measured temperature of 2 K. We observe a large AHE (ρ_*xy*_ of ∼1 μohm·cm) around *T*_N_ comparable in size to common ferromagnets, even though the films show a weak net moment of only 0.008 μ_B_/Mn [arising from a small distortion of the noncollinear Γ_4g_-spin arrangement in the (111) plane]. This large AHE in the absence of a substantial net moment suggests a Berry phase mechanism. The weak moment provides a controllability to switch antiferromagnetic domains and associate Berry curvature with applied magnetic field. Furthermore, we provide direct MOKE imaging and deterministic controllability of the Γ_4g_ antiferromagnetic domains giving rise to large Berry curvature–driven AHE, via a zero-loop Sagnac interferometer. Our findings reveal that antiferromagnetic domains could be switched and saturated with an applied magnetic field of 9 T just below the *T*_N_, whereas they become nearly unswitchable at low temperature. These observations could be explained by the presence of magnetic anisotropy, leading to partial domain pinning as schematically illustrated in Fig. 1C. Notably, we can use magnetic-field cooling to align the antiferromagnetic domains, resulting in saturated AHE at low temperature, indicating precise controllability, which are further supported by theory calculations. These insights advance our understanding of the AHE order in NCAFs and will provide crucial guidance for harnessing AHE in antiferromagnetic-based electronics.

## RESULTS

### High-quality epitaxial Mn_3_NiN thin film

Mn_3_NiN is an antiperovskite with the cation (Mn) and anion (N) positions interchanged compared to typical *AB*O_3_ perovskite. Whereas *AB*O_3_ perovskites can be viewed as alternate stackings of *A*O and *B*O_2_ layers along the [001] direction, Mn_3_NiN can be described as similar stackings of MnNi and Mn_2_N layers (fig. S3). We were able to grow high-quality Mn_3_NiN thin films on (La_0.3_Sr_0.7_)(Al_0.65_Ta_0.35_)O_3_ (LSAT) substrates using optimized growth conditions in a dc reactive magnetron sputtering system, guided by our previous research (*10*, *15*). Out-of-plane x-ray diffraction (XRD) shows a strong Mn_3_NiN peak with distinct Kiessig fringes, indicating high crystalline quality that is further corroborated by the streaky reflection high-energy electron diffraction (RHEED) pattern (figs. S4 and S5). X-ray reciprocal space mapping measurements centered on the asymmetrical ($\bar{1}13$) peak (fig. S4B) indicate that *c/a* = 0.9952 at room temperature. Atomic force microscopy imaging shows an atomically smooth surface with a roughness of ∼0.3 nm (fig. S4C). These data (further details are provided in figs. S4 to S7) establish the high-quality epitaxial growth of single-crystal Mn_3_NiN thin films, allowing conclusive magnetic, transport, and optical measurements.

**Fig. 2.**
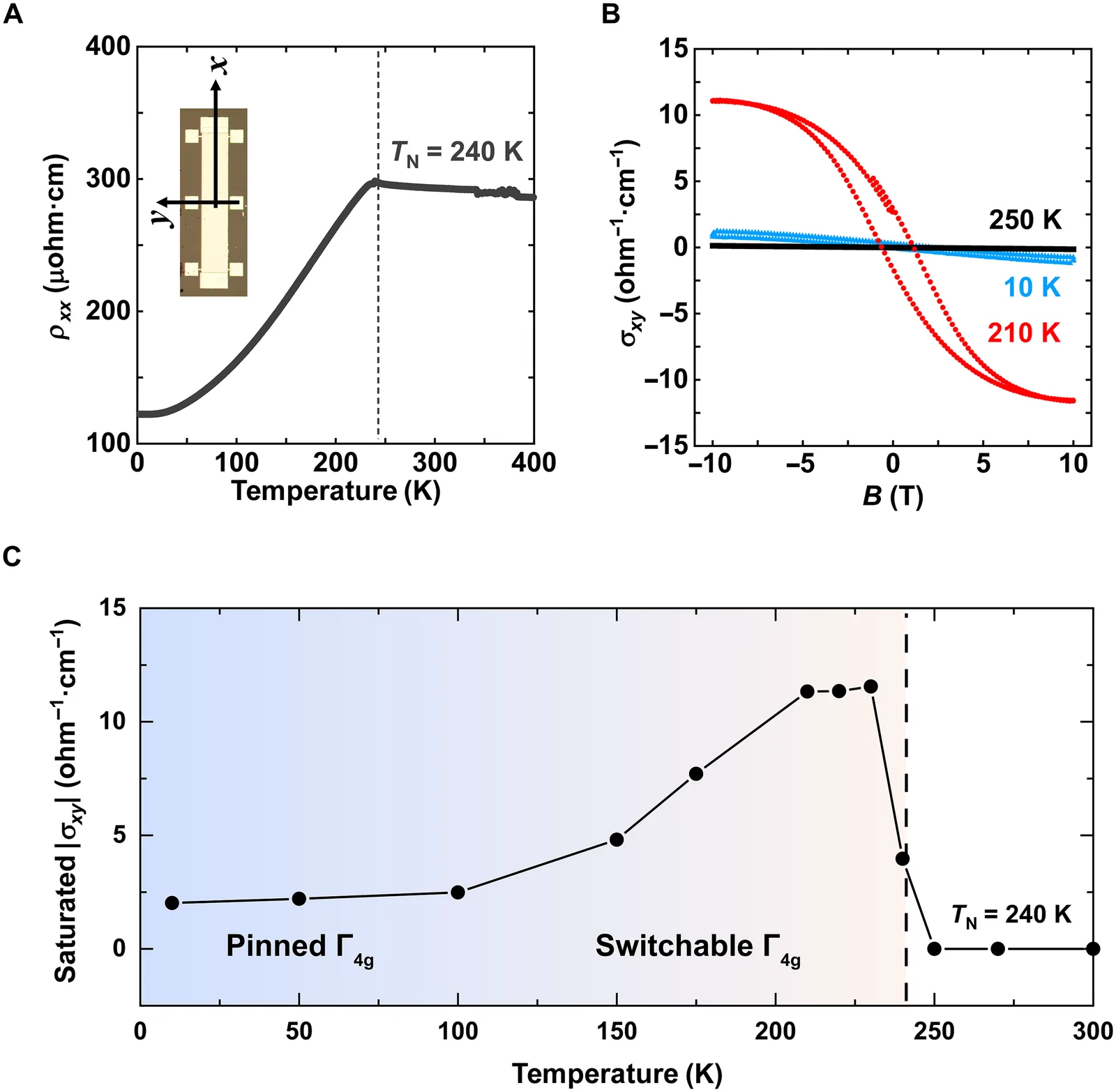
Large AHE in Mn_3_NiN single-crystal thin films. (**A**) Temperature-dependent variation of longitudinal sheet resistivity (ρ*_xx_*) showing excellent metallic behavior below the Néel transition of 240 K. Corresponding optical image of the Hall bar shown in the inset. (**B**) Hall conductivity versus out-of-plane applied magnetic field showing clear anomalous Hall signal below the *T*_N_. (**C**) Temperature dependence of the saturated AHC change, showing maximum around the paramagnetic to Γ_4g_ transition temperature and then gradually decreases and becomes almost zero below ∼100 K.

To investigate the transport properties, we fabricated Hall bar devices on such high-quality Mn_3_NiN epitaxial film (inset to Fig. 2A). The longitudinal resistivity shows a clear kink at the paramagnetic to antiferromagnetic Néel transition of 240 K (Fig. 2A), consistent with our magnetization measurements (fig. S8). The transverse resistivity shows a transition from a linear dependence on magnetic field above *T*_N_, consistent with an ordinary Hall effect, to a highly nonlinear field dependence with a clear hysteresis below *T*_N_ (Fig. 2B), indicating the emergence of an anomalous Hall contribution (*11*). We extracted the AHE resistivity component by subtracting the approximate high-field linear ordinary Hall component from the total Hall signal (fig. S11A), noting that a strictly linear ordinary Hall contribution cannot be uniquely defined and that this procedure provides an estimate of the anomalous Hall component. The resulting AHE component exhibits clear hysteresis with an apparent saturation at high magnetic field, corresponding to near alignment of antiferromagnetic domains, although complete saturation of the spin structure cannot be strictly assumed. The finite Hall signal observed at zero magnetic field after field cycling corresponds to the remanent AHE, reflecting the retained domain configuration. In contrast, in the absence of field history, the net anomalous Hall signal is expected to vanish due to averaging over multiple antiferromagnetic domains. We deduced anomalous Hall conductivity (AHC) from anomalous Hall resistivity (AHR) and sheet resistivity data as

$$\sigma_{\mathrm{xy}}=-\frac{\rho_{\mathrm{xy}}}{\rho_{\mathrm{xx}}^{2}}$$ (1)

The σ_*xy*_ of a 40-nm Mn_3_NiN thin film exhibits an unusual temperature dependence with a maximum value of about ∼11 ohm^−1^·cm^−1^ (Fig. 2C) and AHR ρ_*xy*_ of ∼1 μohm·cm (fig. S10). This behavior was independent of thickness as a similar result was obtained on a 20-nm thin film, excluding any role of the surface magnetization contribution (fig. S12) (*29*). The large intrinsic value of anomalous Hall is of immense interest and importance, as it is comparable to common ferromagnetic metals such as 0.6 μohm·cm for Fe with net moment 2.18 μ_B_/Fe, 0.1 μohm·cm for Co with moment 1.67 μ_B_/Co, and 0.15 μohm·cm for Ni with moment 0.62 μ_B_/Ni (*30*–*34*). The 0.008 μ_B_/Mn magnetic moment of our Mn_3_NiN films (fig. S8), is too small to generate large σ_*xy*_ and ρ_*xy*_ (*11*) in our Mn_3_NiN thin films like conventional mechanisms in ferromagnets. These large σ_*xy*_ and ρ_*xy*_ of Mn_3_NiN films are observed in various thicknesses and comparable to those found in other noncollinear antiferromagnets (figs. S13 and S15 and table S1), which is consistent with a nonvanishing Berry curvature from the Γ_4g_ spin structure (*14*, *35*, *36*). Thus, we conclude that the effect of the net magnetic moment is secondary in generating the observed AHE. Comparing the anomalous behavior of σ_*xy*_ with the net moment (figs. S16 and S17) further supports this conclusion.

The temperature-dependent response of anomalous σ_*xy*_ exhibits nontrivial trend. While the σ_*xy*_ is zero above *T*_N_, it increases to large values (∼11 ohm^−1^·cm^−1^) immediately below *T*_N_, before gradually dropping close to zero as temperature is further decreased below ∼100 K. We further note that the evolution of the hysteresis and coercive field with temperature suggests thermally activated domain dynamics, where domain reorientation becomes progressively more difficult upon cooling and remains only weakly field-responsive at low temperatures. One possible explanation for this unusual gradual suppression of σ_*xy*_ upon cooling is that the system partially evolves toward Γ_5g_ magnetic configuration upon cooling. However, the transition may remain incomplete in strained thin films, resulting in a partially rotated magnetic configuration rather than a fully developed Γ_5g_ phase. In this scenario, the reduction of σ_*xy*_ reflects the progressive rotation of spins toward Γ_5g_, where the net Berry curvature integrated over the Brillouin zone for all occupied states approaches zero, rather than a complete disappearance of Berry curvature itself. This interpretation is consistent with prior observations in bulk polycrystalline Mn_3_NiN and thin film Mn_3_NiN and Mn_3_Pt by other groups (*13*, *22*, *36*). At the same time, our neutron diffraction measurements (figs. S8 and S9) confirm the presence of long-range magnetic order and are consistent with a substantial Γ_4g_ component persisting down to low temperatures. We therefore emphasize that our data support a partial rotation toward Γ_5g_ rather than a complete phase transition. We note that the evolution of magnetic peak intensity with decreasing temperature may reflect both changes in magnetic configuration and the strengthening of the order parameter due to reduced thermal fluctuations, and therefore is not conclusive on its own. Such discrepancy could arise from differences in growth methods and strain states of materials, where further microscopic probes, e.g., additional magnetic reflections in neutron diffraction or strain-dependent DFT calculations (*17*), would be valuable to fully resolve the magnetic ground state in epitaxial Mn_3_NiN thin films. Another possibility could be the formation of antiferromagnetic domains (*21*), where equal population of domains of opposite polarity could cancel, leading to zero σ_*xy*_ from the entire sample. Thus, to have a holistic understanding of the origin of such unique temperature dependence of anomalous Hall, the direct observation of antiferromagnetic domains dynamics is of high importance to advance the research on emergent noncollinear antiferromagnetic spintronics.

### Sagnac MOKE imaging of antiferromagnetic domains

**Fig. 3.**
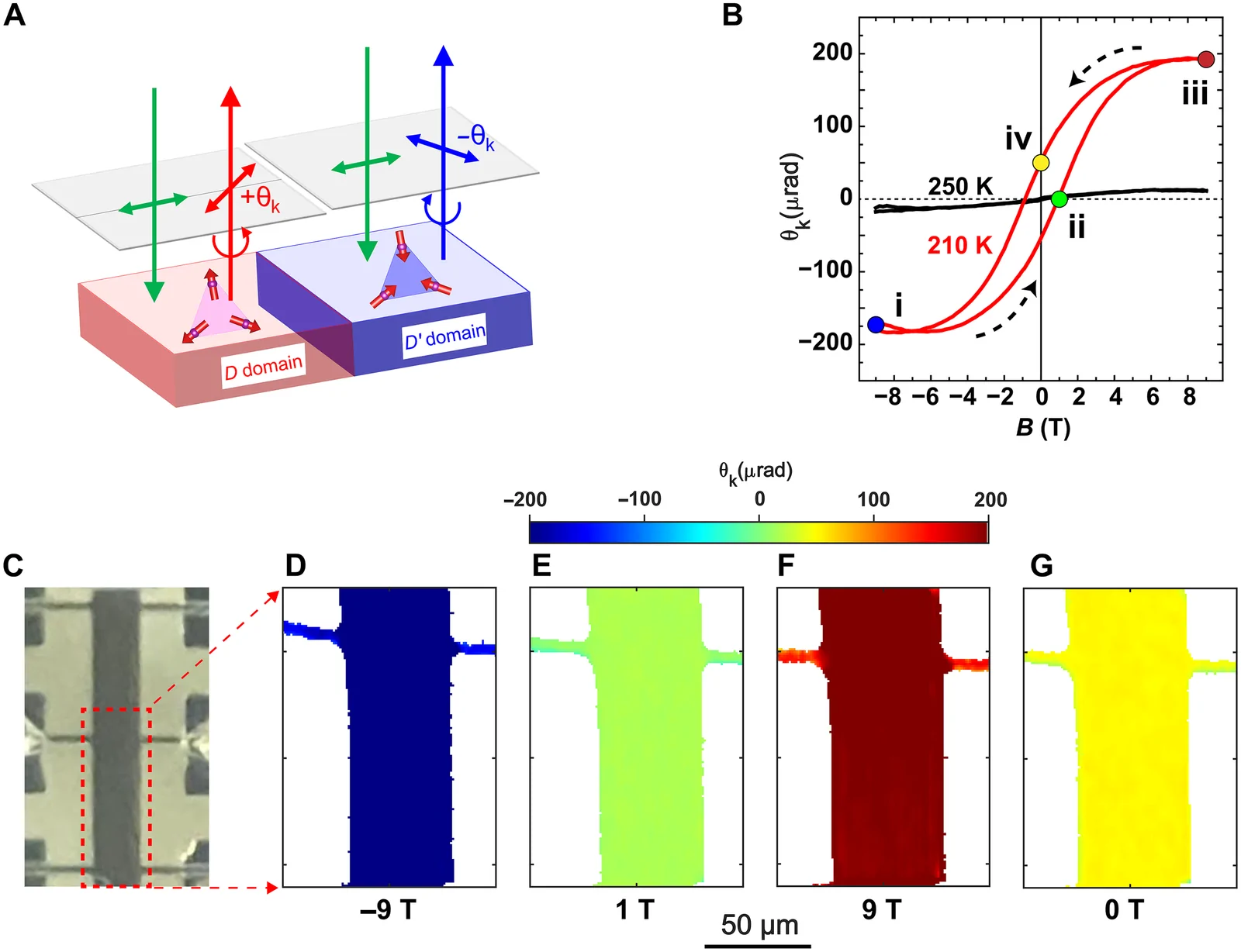
Direct Sagnac MOKE imaging of magnetic domain switching in Mn_3_NiN thin films. (**A**) Schematic illustration of magneto-optical response corresponding to different types of Γ_4g_ domains in Mn_3_NiN, leading to positive or negative Kerr rotations. (**B**) Kerr rotation θ_k_ versus out-of-plane applied magnetic field at 250 K (above *T*_N_) and 210 K (below *T*_N_), showing a pronounced hysteresis loop at the latter temperature with a saturated Kerr signal of θ_k_ ≈ 200 μrad, and remnant θ_k_ similar in magnitude to the value in extended data Fig. 4A, with a nearly flat linear response at 250 K. (**C**) Optical images of Mn_3_NiN Hall bar device. (**D** to **G**) Scanning Kerr imaging taken at multiple field values during a hysteresis magnetic field sweep, corresponding to the points (denoted by i, ii, iii and iv) marked in the hysteresis loop in (B).

MOKE imaging can directly reveal Berry curvature and antiferromagnetic domain structures, as previously demonstrated in Mn_3_Sn (*21*), where the Kerr rotation angle θ_k_ serves as a local probe of σ_*xy*_ at optical frequencies (Fig. 3A). The antiferromagnetic domain imaging had also been demonstrated using scanning anomalous Nernst microscopy and nanoscale nitrogen-vacancy magnetometry (*24*, *37*, *38*). These techniques provide complementary information on domain structure and magnetization distribution; however, they do not directly establish a quantitative link between local Kerr response and electrical transport within the same device geometry. In this work, we use a scanning fiber-optic Sagnac interferometer, capable of detecting Kerr rotation with high-resolution nanoradian sensitivity (fig. S18), integrated into a scanning optical probe for micrometer-resolution imaging of Kerr rotation, with spot size (∼1.5 μm), pixel size (∼1 μm), Kerr sensitivity (∼10 nrad), and more surface sensitivity compared to bulk properties characterized by magnetic and transport measurements, as described in previous publications (*28*, *39*, *40*). This ultrahigh sensitivity enables detection of very small Berry curvature–related signals that are otherwise inaccessible with conventional techniques. Unlike conventional MOKE or AHE techniques, which often rely on magnetic hysteresis to isolate intrinsic signals, the Sagnac interferometer’s time-reversal symmetric design inherently suppresses nonreciprocal artifacts such as optical misalignment and extrinsic scattering, enabling precise, field-stable measurements of θ_k_. We perform Sagnac MOKE imaging and single-point measurements across a range of temperatures and magnetic fields (Fig. 3B and fig. S19A) on Hall bar devices (Fig. 3, C to G), capturing the domain evolution and enabling direct correlation with transport signatures of Berry curvature. This approach allows us to track the evolution from field-switchable domain configurations near *T*_N_ to strongly pinned, weakly field-responsive domain configurations at low temperatures, thereby directly linking spatial domain behavior with transport responses.

We begin by examining the temperature dependence of the Kerr rotation θ_k_. Field-cooled (FC) measurements of θ_k_ at fixed points on the sample surface during a warm-up in zero field reveal a sharp onset of ≈60-μrad Kerr rotation below *T*_N_, which remains nearly constant down to 2 K (fig. S19A). This temperature-independent behavior of θ_k_ is consistent with the magnetization and neutron data; however, it contrasts sharply with the strong temperature dependence of the AHC σ_*xy*_ (Fig. 2C), suggesting that the underlying magnetic order remains stable while the transport response evolves. This direct comparison highlights a key advantage of the Sagnac MOKE technique, which selectively probes the intrinsic Berry curvature–related response through Kerr rotation.

To probe the temperature evolution of the spatial distribution of antiferromagnetic domains (driving the Berry curvature) on the sample surface, we first repeat the process of cooling the sample under +0.3 T applied magnetic field to polarize all domains before removing the field and performing scanning images of θ_k_ in zero field, both above and below *T*_N_ (fig. S19B). At 300 K, a scan of θ_k_ shows a uniform and near zero value across the entire sample, consistent with the absence of net Berry curvature. In contrast, at 2 K, we observe a strong, spatially homogeneous Kerr response across the entire area, indicating a coherent alignment of antiferromagnetic domains. These results indicate that the Γ_4g_-derived magnetic order remains robust at low temperatures, although some domains with a partial rotation toward the Γ_5g_ configuration cannot be excluded. However, the mismatch between the static almost uniform Kerr response (fig. S19) and the evolving σ_*xy*_ (Fig. 2) highlights that domain dynamics alone cannot fully account for the unusual temperature dependence of the AHE in this system.

**Fig. 4.**
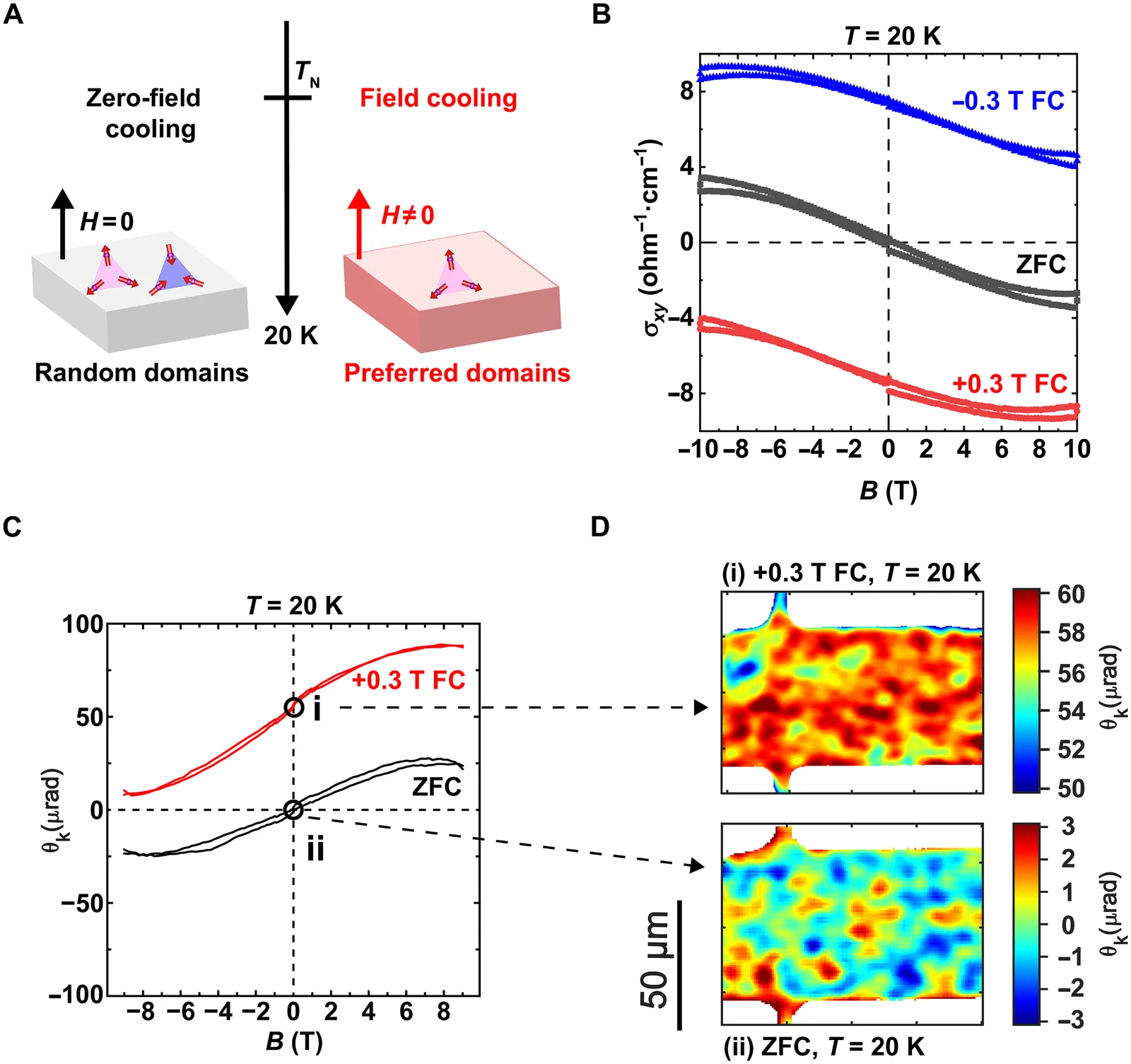
Direct control of Berry curvature by applied poling magnetic field. (**A**) Schematic illustration of the antiferromagnetic Γ_4g_ domain alignment under zero or finite out-of-plane magnetic field cooling conditions in Mn_3_NiN thin film. (**B**) Variation of the AHC with magnetic field while ramping the field from 0 to 10 T at 20 K, after cooling the sample under ZFC and ±0.3 T. (**C**) Kerr rotation θ_k_ as a function of magnetic field under the same cooling conditions as in (A), directly measured using a Sagnac interferometer. The ZFC curve exhibits a symmetric hysteresis with no remnant θ_k_, while the +0.3 T FC curve shows a robust remanent Kerr signal at zero field, indicating strong domain alignment. (**D**) Corresponding scanning Kerr images at 0 T following ZFC and 0.3 T field cooling, revealing a disordered domain landscape with spatially random θ_k_ contrast centered around zero (±3 μrad) and a strongly polarized domain configuration with large, positive θ_k_ across the device, respectively.

To further investigate the role of antiferromagnetic domains and their switching behavior in Mn_3_NiN, we performed magnetic field–dependent MOKE measurements at 250 and 210 K (Fig. 3B), along with scanning at key points along the 210 K hysteresis loop, including positive and negative saturation fields (±9 T), the coercive field, and zero field (Fig. 3, D to G). At 210 K, well below the magnetic transition, we observe a pronounced Kerr hysteresis loop that reaches a saturated θ_k_ of ≈200 μrad below 9 T, on scale with the largest reported Kerr rotation in NCAFs (*21*). The field dependence at 250 K remains nearly linear, with nominal variations representing a paramagnetic response above *T*_N_. At 210 K, both the AHC and remnant θ_k_ reach magnitudes comparable to those of Mn_3_Sn (*21*). A remnant θ_k_ of ≈54 μrad persists at zero field after sweeping back down from +9 T, indicating the equivalence of +0.3 T field cooling (fig. S19A), and fully polarizing the sample at 210 K with a 9-T field. Weak spatial variations in Kerr signal are noticeable in Fig. 3 (D to G), particularly between Fig. 3 (F and G), which possibly originate from the presence of multiple small magnetic domains contributing to the larger domain landscape within the Hall bar. In Fig. 3 (D to G), a relatively wide color scale was used to cover the full Kerr signal range observed across different applied magnetic fields. This broader color scale visually suppresses the contrast of smaller domain variations, making the images appear more similar than they are. The underlying domain structure responsible for this variation is more clearly visible in Fig. 4D, where a narrower color scale enhances the contrast.

Kerr imaging at selected field values during the 210-K hysteresis measurements reveals key insights into the switching mechanism. The switching of antiferromagnetic domain polarization proceeds through an apparently uniform evolution across the entire 50 μm–by–150 μm area of the Hall bar (Fig. 3C). From −9 to +9 T, the polarization transitions coherently between ±200 μrad, without visible evidence of domain wall motion at this scale. The findings in Fig. 3 (C to G) indicate that the domain reversal mechanism in Mn_3_NiN is predominantly coherent over macroscopic length scales, but may involve fine-scale domain evolution at the mesoscopic or microscopic level. Therefore, we perform higher resolution scans in the following section to test for potential subtle spatial variations in θ_k_, which reveal the presence of weak domain textures not resolved in the coarse scans in Fig. 3 (C to G), and also try to rule out the inconsistent transport response at low temperature.

### Understanding the temperature-dependent AHE

We find that Mn_3_NiN exhibits a large AHC near the *T*_N_, which progressively diminishes upon further cooling and ultimately vanishes below 100 K. This behavior allows us to define two distinct regimes: region I (∼100 K < *T* < 240 K), where a notable AHC is observed, and region II (*T* < ∼100 K), where the AHC is essentially diminished. To elucidate the microscopic origin of this unusual temperature dependence of σ_*xy*_ (Fig. 2C), we use Sagnac MOKE as a deterministic probe of Berry curvature domains and their coupling to the underlying Γ_4g_ magnetic domains. We cooled Mn_3_NiN thin films from high temperature through the *T*_N_ to 20 K under both zero-field cooling (ZFC) and FC conditions and then measured both AHC and θ_k_ as a function of magnetic field (Fig. 4A).

First, we made Hall measurements at 20 K (region II) after cooling the sample at positive, zero, and negative applied fields (Fig. 4B). Such a poling under an applied magnetic field leads to significant pinning of antiferromagnetic domains at low temperature, and thus, the sign of anomalous Hall is not switchable (Fig. 4B), likely due to increased magnetic anisotropy. Notably, the ZFC Kerr hysteresis loop (Fig. 4C, black curve) closely mirrors the AHE behavior (Fig. 4B and fig. S17), exhibiting zero remnant θ_k_ under ZFC conditions. Thus, in the absence of field history, the net AHE averages to zero due to multidomain averaging of Γ_4g_ magnetic domains. The weak linear field dependence observed in both Kerr and transport measurements is consistent with a small uncompensated moment or canting contribution, rather than a paramagnetic response. Such weak moments can arise from domain boundaries, strain-induced canting, and slight noncollinearity deviations within domains, which also deserve detailed characterizations and understanding for future study. In contrast, the FC loop (Fig. 4C, red curve) shows a finite remnant θ_k_ at zero field, unchanged from the remanent Kerr signal acquired during the +0.3-T field cooling, indicating that the field cooling can effectively align the antiferromagnetic domain (driving the Berry curvature) beforehand. However, similar to the AHE measurements, the sign of the remnant ZFC signal cannot be reversed, even by sweeping field to ±9 T, indicating that the antiferromagnetic domains are pinned in and robust against switching at low temperatures. This behavior indicates that antiferromagnetic domains become increasingly difficult to reorient at low temperatures, leading to a stable remanent state.

The nearly unswitchable nature of the domains at low temperature behavior suggests that a net Berry curvature emerges only when domains are aligned during cooling, or by large magnetic fields at sufficiently high temperatures. We further verified this by a full temperature range FC and zero-field warming transport measurement on Mn_3_NiN. We observe the nonvanishing σ_*xy*_ also down to 2 K (fig. S20), which indicates that the FC-induced domain alignment is maintained below *T*_N_ (but nonswitchable at region II) and is consistent with a partially rotated magnetic state rather than a complete transition to Γ_5g_. Notably, the σ_*xy*_ exhibits a clear temperature dependence upon warming (fig. S20), decreasing gradually despite the initial FC domain alignment. This behavior indicates that the anomalous Hall response contains both intrinsic Berry curvature contributions and extrinsic scattering contributions (e.g., skew scattering), with the latter being more sensitive to temperature. In contrast, the Kerr rotation remains nearly temperature independent below *T*_N_, reflecting its dominant sensitivity to the intrinsic Berry curvature–related electronic structure (*41*). In addition, the remanent σ_*xy*_ and θ_k_ signals remain stable for many hours without measurable decay (fig. S21), further verifying a robust domain configuration below the *T*_N_, giving a potential for permanent antiferromagnetic storage devices. These observations demonstrate that at low temperatures (region II in Fig. 3), antiferromagnetic domains become pinned and cannot be reconfigured by magnetic field alone, explaining the near-zero AHE seen under ZFC conditions in Fig. 2C.

To directly visualize this domain-pinning behavior, we performed scanning Kerr imaging at 20 K after cooling under various field conditions, fully using the ∼1-μm resolution of the scanning Sagnac probe. A θ_k_ scan taken after ZFC to 20 K (Fig. 4D) reveals a disordered domain landscape with spatially random but near-zero θ_k_ in the range of ±3 μrad, consistent with a vanishing net Berry curvature composed of weak, locally uncompensated domains. Assuming a random formation of fully saturated domains in the ZFC state, we can estimate the domain size as

$$d=\frac{\sigma w}{\theta_{\text{sat}}}=\frac{2\;\text{μrad}\;\times \;1.5\;\text{μm}}{200\;\text{μrad}}=15\;\text{nm}$$ (2)

where σ, *w*, θ_sat_ are the standard deviation, beam width, and saturated Kerr rotation from Fig. 3B, respectively. Notably, the ZFC θ_k_ scan images taken at 20 K, after cooling in a +0.3-T field (Fig. 4D), both show Berry curvature landscapes that are indistinguishable from each other. These higher-resolution scans reveal fine domain textures with a variation of ±4 μrad from the polarized θ_k_ value that were not resolved in the earlier, zoomed-out images collected during the hysteresis loop (Fig. 3, D to G). In those coarse scans, the large color scale and broader spatial averaging masked subtle variations in θ_k_, making the Berry curvature landscape appear uniformly polarized. Here, the more sensitive imaging exposes the underlying domain structure, confirming that the apparent coherence in the previous measurements arises from macroscopic averaging over a multidomain state.

Together, these data provide a microscopic explanation for the suppressed σ_*xy*_ at low temperature (region II in Fig. 3) under ZFC: The antiferromagnetic domains (driving the Berry curvature) are dominantly present but randomly oriented canceling out the macroscopic signal. In addition, the Kerr rotation measurements as a function of temperature reveal that θ_k_ remains nearly constant below the *T*_N_, suggesting that the underlying Berry curvature persists to the lowest measured temperatures, even as the AHE tends to diminish under ZFC. The scanning MOKE images in Fig. 4D confirm that the same Berry curvature landscape probed in both the anomalous transport σ_*xy*_ (Fig. 2B) and Kerr hysteresis loop measurements respectively at 210 K (Figs. 2B and 3B) exists down to low temperatures regardless of field cooling. This behavior confirms that the loss of anomalous Hall response at low temperature arises not from the suppression of Berry curvature itself, but from the inability to reorient the pinned domains below 210 K with the available magnetic field strength.

The ability to stabilize and control domain polarization through field cooling across *T*_N_ highlights the importance of thermal history and magnetic field in orienting the domains in Mn_3_NiN. The high sensitivity of the zero-area-loop Sagnac interferometer is essential for detecting these weak, domain signals in zero field; without it, the presence of dominant pinned antiferromagnetic domains might have remained hidden.

### Theoretical model of domain switching

**Fig. 5.**
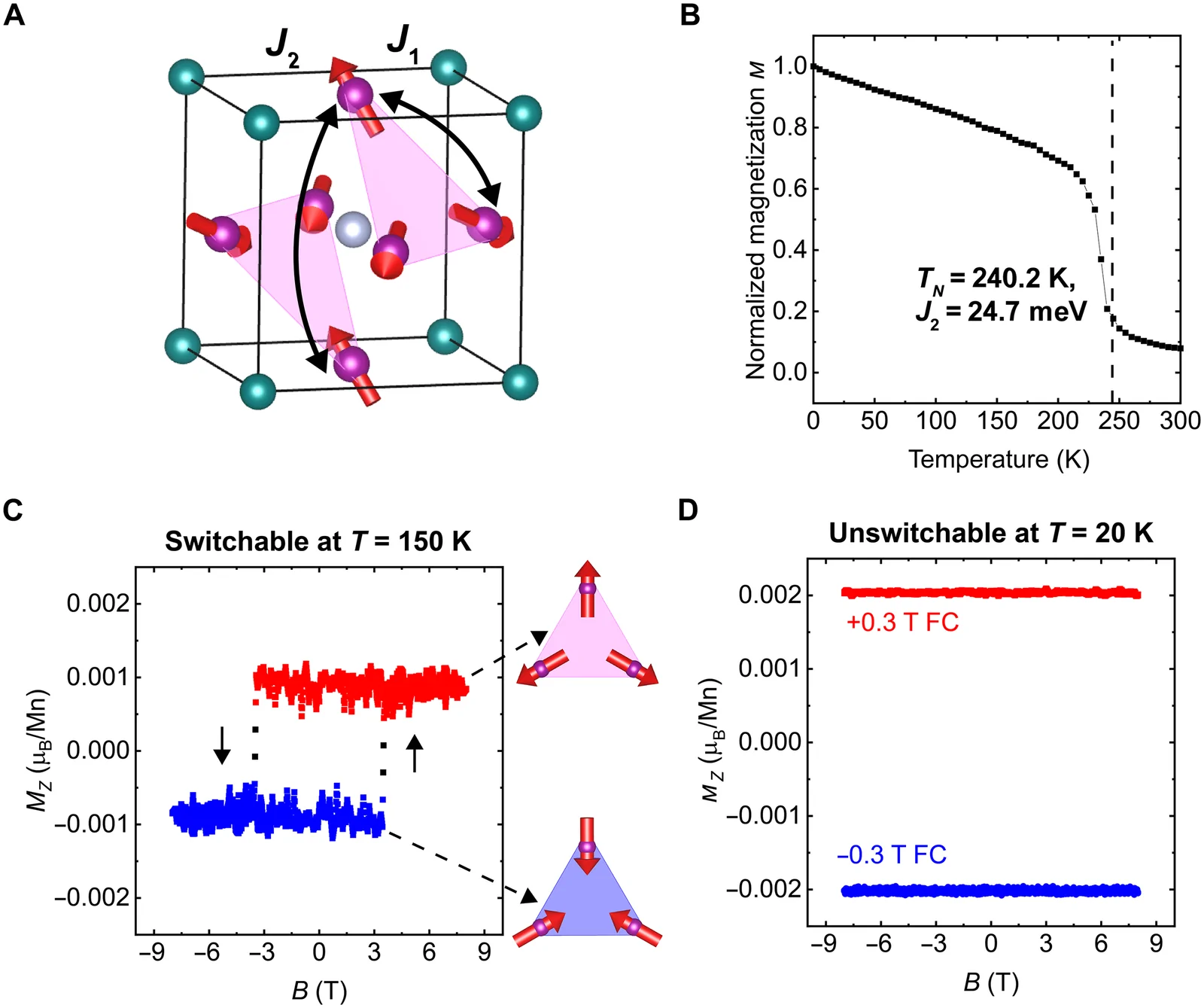
Understanding of the magnetic domain dynamics via theoretical simulations of Mn_3_NiN thin films. (**A**) Schematic illustration of the nearest and next nearest neighbor exchange coupling considered for calculations in Mn_3_NiN. (**B**) Simulation of temperature-dependent response of bulk magnetization, clearly showing the Néel transition at ∼240 K, which is in agreement with the experimental observations. (**C**) Corresponding calculation of the out-of-plane net magnetization switching versus applied magnetic field at 150 K, where the Γ_4g_ domain could be easily aligned and switched with Γ_4g_ spin configuration. (**D**) Simulations of the out-of-plane net magnetization at 20 K after different field cooling, displaying strong nonswitchability of the Γ_4g_ magnetic domains, agreeing with the experimental data. Domains can only be poled to write information by applied field from a temperature above *T*_N_.

To elucidate the magnetic domain switching of Mn_3_NiN with the Γ_4g_ spin ordering as a function of temperature, we perform first-principles and atomistic spin-dynamics simulations, as described in Supplementary Text. The latter is based on a Heisenberg Hamiltonian that includes exchange interactions between first and second nearest-neighbor Mn atoms (Fig. 5A), as well as single-ion magnetic anisotropy. Figure 5B shows the calculated temperature dependence of the magnetization, in good agreement with the experiments.

We examine two scenarios of spin ordering at low (20 K) and high (150 K) temperatures by calculating the out-of-plane component of the net magnetic moment *M*_net⊥_. The sign of this moment is uniquely linked to *D* and *D*′ types of Γ_4g_ antiferromagnetic domains, and its reversal implies domain switching. To probe this behavior, we first computationally perform field cooling to stabilize either the *D* or *D*′ domain and then compute the hysteresis curve *M*_net⊥_(*B*) at a given temperature. We find that at 150 K, *M*_net⊥_ exhibits clear field-induced switching within the applied magnetic field range of −8 to 8 T (Fig. 5C). In contrast, at 20 K, it shows two distinct nonswitchable responses (Fig. 5D). This low-temperature behavior is consistent with a regime where spins evolve toward a partially rotated configuration approaching Γ_5g_, leading to reduced field responsiveness.

The large single-ion magnetic anisotropy is the primary factor responsible for the robust pinning of antiferromagnetic domains at low temperatures. At 20 K, thermal fluctuations are insufficient to overcome the anisotropy barrier separating the *D* and *D*′ domains, resulting in nonswitchable domain configurations. In this regime, the system does not undergo a complete transition to Γ_5g_ but rather exhibits a partially rotated magnetic state that reflects an evolution toward the Γ_5g_ ground state. At 150 K, however, thermal fluctuations promote thermally assisted crossing of this barrier, enabling field-induced switching between *D* and *D*′ domains (Fig. 1C). This behavior can be equivalently viewed as a temperature-induced reduction of the effective uniaxial anisotropy energy, arising from thermal averaging over spin fluctuations. Such renormalization of the anisotropy is qualitatively consistent with the Callen-Callen power-law scaling (*42*–*44*).

These theoretical results are fully consistent with our transport and Sagnac imaging measurements, which show that Γ_4g_ domains can be aligned during field cooling but remain nearly unswitchable below ≲100 K, while becoming easily switchable only at elevated temperature, consistent with a progressive rotation of spins toward Γ_5g_ without a complete phase transition.

## DISCUSSIONS

We have demonstrated the growth of high-quality epitaxial antiperovskite Mn_3_NiN single-crystal thin films on perovskite LSAT substrates with NCAF Γ_4g_ phase and have shown its Berry-phase driven AHE (σ_*xy*_) through conventional magnetic and transport characterizations. While the Γ_4g_ ordering remains dominant, our combined experimental and theoretical results indicate a temperature-driven evolution toward a partially rotated state approaching Γ_5g_ at low temperatures. Furthermore, through Sagnac MOKE measurement, we directly imaged and precisely controlled the antiferromagnetic domains (driving the Berry curvature) and AHE in Mn_3_NiN by temperature and magnetic field, explained by theoretical calculations. These results not only provided insight into the Berry curvature nature of Mn_3_NiN, but also explained the unusual temperature-dependent AHE behavior, revealing a path toward AHE-based spintronic systems.

Looking beyond Mn_3_NiN, it would be valuable to investigate other potential NCAF systems using a Sagnac microscope, where current-induced switching of AHE and antiferromagnetic tunnel junction (*7*, *8*) showed substantial promise toward spintronic applications. As the effective MOKE imaging intensively provides nanoscale probing of Berry curvature and antiferromagnetic domain control in these NCAFs, experimental testing of these scenarios would be an interesting direction for future research.

## MATERIALS AND METHODS

### Thin-film growth and characterizations

Epitaxial 40-nm Mn_3_NiN single-crystal thin films were grown on (001)-oriented LSAT substrates at 650°C under an Ar/N_2_ (78%:22%) mixed atmosphere of 10 mTorr by dc reactive magnetron sputtering using a stoichiometric Mn_3_Ni target, which was monitored by in situ RHEED. Mn_3_NiN films were measured by atomic force microscopy and XRD spectroscopy for structural characterization.

### Magnetic and transport measurements

dc magnetic measurements were carried out in a SQUID magnetometer on a 3 mm–by–3 mm sample, and all magneto transport measurements were conducted in a Physical Property Measurement System on patterned Hall bar (50 μm by 200 μm) with Ti/Pt electrodes (first Ti was deposited, which acts as a binder to the subsequent Pt electrodes).

### Neutron diffraction

Single crystal neutron diffraction measurements were performed on the WISH time-of-flight diffractometer at ISIS, the UK neutron and muon source. Approximately 250-nm-thick (001) Mn_3_NiN film samples with lateral dimensions of 10 mm by 8 mm were oriented for the measurement of nuclear and magnetic diffraction intensities in the (HK0) reciprocal lattice plane. The sample was mounted within a ^4^He cryostat, and diffraction patterns were collected from a base temperature of 1.5 K up to 250 K.

### Sagnac MOKE measurements

MOKE measurements were performed using a zero-loop fiber-optic Sagnac interferometer and microscope as described in our earlier publications (*28*, *39*, *40*). As shown in the schematics (fig. S18) in (*28*), the beam of light is routed by a fiber circulator to a fiber polarizer. After the polarizer, the polarization of the beam is at 45° to the axis of a fiber-coupled electro-optic modulator (EOM), which generates 4.6-MHz time-varying phase shifts $\phi_{m}\text{sin}\left(\omega t\right)$, where the amplitude ϕ_m_ = 0.92 rad between the two orthogonal polarizations that are then launched into the fast and slow axes of a polarization-maintaining (PM) single-mode fiber. Upon exiting the fiber, the two orthogonally polarized linearly polarized beams are converted into right- and left-circularly polarizations by a quarter-wave plate (QWP) and are then focused onto the sample. After reflection from the sample, the same QWP converts the reflected beams back into linear polarizations with exchanged polarization axes. The two beams then pass through the PM fiber and EOM but with exchanged polarization modes in the fiber and the EOM. At this point, the two beams have gone through the same path but in opposite directions, except for a phase difference of ∆φ from reflection off the magnetic sample and another time-varying phase difference by the modulation of EOM. This nonreciprocal phase shift ∆φ between the two counterpropagating circularly polarized beams upon reflection from the sample is twice the Kerr rotation ∆φ = 2θ_K_. The two beams are once again combined at the detector and interfere to produce an optical signal P(t)

$$P\left(t\right)=\frac{1}{2}\;P\left\{1+\text{cos}\left[∆\varphi +\phi_{m}\text{sin}\left(\omega t\right)\right]\right\}$$ (3)

where *P* is the returned power if the modulation by the EOM is turned off. For MOKE signals that are slower that the 4.6-MHz modulation frequency used in this experiment, we can treat ∆φ as a slowly time-varying quantity. P(t) can be further expanded into Fourier series with the first few orders listed below

$$\begin{array}{c} \frac{P\left(t\right)}{P}=\frac{1}{2}\;\left[1+J_{0}\left(2\phi_{m}\right)\right]+\left[\text{sin}\left(∆\varphi \right)J_{1}\left(2\phi_{m}\right)\right]\;\text{sin}\left(\omega t\right)\\ +\left[\text{cos}\left(∆\varphi \right)J_{2}\left(2\phi_{m}\right)\right]\;\text{cos}\left(2\omega t\right)+2\;J_{3}\left.\left(2\phi_{m}\right)\right.\;\text{sin}\left(3\omega t\right)+\ldots \end{array}$$ (4)

where $J_{1}\left(2\phi_{m}\right)$ and $J_{2}\left(2\phi_{m}\right)$ are Bessel *J* functions. Lock-in detection was used to measure the first three Fourier components: the average (dc) power (*P*_0_), the first harmonics (*P*_1_), and the second harmonics (*P*_2_). The Kerr rotation can then be extracted using the following formula

$$\theta_{K}=\frac{1}{2}∆\varphi =\frac{1}{2}{\text{tan}}^{-1}\left[\frac{J_{2}\left(2\phi_{m}\right)P_{1}}{J_{1}\left(2\phi_{m}\right)P_{2}}\right]$$ (5)

### DFT calculations

First-principles DFT with fully relativistic ultrasoft pseudopotentials (*45*) was performed using Quantum ESPRESSO (*46*). The exchange and correlation effects were treated within the generalized gradient approximation (*47*). The plane-wave cutoff energy of 52 Ry and a 16 × 16 × 16 *k*-point mesh in the irreducible Brillouin zone were used in the calculations. The experimentally measured in-plane lattice parameter *a* = 3.899 Å of Mn_3_NiN was used in the calculations. Spin-orbit coupling and noncollinear Γ_4g_ antiferromagnetism of Mn_3_NiN were included in the calculations. The AHC was calculated using the PAOFLOW code (*48*) based on pseudo-atomic orbitals (PAOs) (*49*, *50*).The adaptive broadening method with a 48 × 48 × 48 *k*-point mesh was used in the calculations.

### Atomistic spin model simulations

Atomistic spin dynamics simulations of Mn_3_NiN are performed using VAMPIRE package (*51*) using its adaptive Monte-Carlo based integrator (*52*) with temperature-dependent magnetization dynamics (*44*) using the following Heisenberg Hamiltonian

$$H=-\frac{J_{1}}{2}\sum_{<\mathrm{ij}>}S_{i}.S_{j}-\frac{J_{2}}{2}\sum_{\ll \mathrm{ij}\gg }S_{i}.S_{j}-k\sum_{i}{\left(S_{i}.e_{i}\right)}^{2}$$ (6)

Here, ***S***_*i*_ is the unit vector of the spin moment at site *i*, $J_{1\left(2\right)}$ is the magnetic exchange interaction energy between first (second) nearest neighbors, with <*ij*> (≪ij≫) indicating a summation over all first (second) nearest neighbors, *k* is the single ion anisotropy constant, and ***e***_*i*_ is a unit vector normal to the cubic face where each site resides. The values used for the magnetic exchange parameters *J*_1_ and *J*_2_ and anisotropy constant *k* are found to be −23.9, 24.7, and 0.133 meV (see text S5 for details). The time step was set to be 1 fs, and periodic boundary conditions along *x*, *y*, and *z* directions were used for all atomistic simulations. For *T*_N_ simulations, a system of 12 × 12 × 12 unit cells was used with 100,000 time steps and temperature increment of 10 K. For net moment isothermal hysteresis loops, a system of 25 × 25 × 25 unit cells were subject to FC + 0.3 T with temperature going from 300 to 0 K to obtain the appropriate domain. Then, the system was heated up to the required temperature for each loop to calculate the hysteresis loop with 1,000,000 time steps at each magnetic field with an increment of 25 mT going from 8 to −8 T, and back to 8 T.

## Supplementary Materials

### This PDF file includes:

Supplementary Text Figs. S1 to S22 Table S1 References
