# Supplementary material for: Direct imaging Berry curvature–induced transport and domain behavior in noncollinear single-crystal antiferromagnetic thin films
Source: Sci Adv. 2026 Aug 26;12(35):eaeb6478. doi: 10.1126/sciadv.aeb6478 (PMC13510672; doi:10.1126/sciadv.aeb6478)
Supplement: Supplementary file 1 [file sciadv.aeb6478_sm.pdf]

Science Advances
AAAS

Supplementary Materials for

Direct imaging Berry curvature–induced transport and domain behavior in noncollinear single-crystal antiferromagnetic thin films

Yuchuan Yao *et al.*

Corresponding author: Jing Xia, xia.jing@uci.edu; Chang-Beom Eom, ceom@wisc.edu

Sci. Adv. **12**, eaeb6478 (2026)
DOI: 10.1126/sciadv.aeb6478

This PDF file includes:

Supplementary Text
Figs. S1 to S22
Table S1
References

Supplementary Text

Structural characterization of Mn_3NiN thin films

A strong Mn_3NiN peak is observed along with distinct Kiessig fringes, indicating high crystalline quality and a pristine interface with the substrate, as shown in fig. S4A and fig. S5 for out of plane scan around (002) peak and full range scan. This is further corroborated by the streaky RHEED pattern after growth and X-ray reflectivity data as shown in the inset to fig. S4A and fig. S6B. The narrow 0.029° full width at half maximum (FWHM) of the rocking curve for Mn_3NiN (002) (fig. S6A) further supports high crystalline quality. The phi scans on the asymmetric $(\bar{1}13)$ peak of Mn_3NiN and LSAT, shown in fig. S7, indicate the in-plane cube-on-cube epitaxial relationship. X-ray reciprocal space mapping (RSM) measurements centered on the asymmetrical $(\bar{1}13)$ peak, shown in fig. S4B, indicate that *c/a*=0.9952 at room-temperature. Atomic force microscope imaging shows an atomically smooth surface with a roughness of ~0.3 nm (fig. S4C). All these data strongly indicate high-quality epitaxial growth of Mn_3NiN thin films, for which magnetic and transport measurements can probe fundamental properties.

Magnetic characterization of Mn_3NiN thin films

Fig. S8A shows temperature dependence of the out-of-plane magnetic moment. From the variation of magnetization with temperature, we clearly note a deviation between field-cooling (FC) and zero-field-cooling (ZFC) curves below the Néel temperature of 240 K (note fig. S10A for in-plane magnetic moment). A field-cooled magnetic moment of ~8 mμ_B/Mn indicates the presence of a weak ferromagnetic moment, like that observed in other non-collinear AFM materials. Isothermal magnetic field sweeps above and below the Néel temperature (fig. S8B) show hysteretic behavior at 230K (below T_N) with a weak saturation moment consistent with the field-cooled measurement of fig. S8A, and nonhysteretic behavior at 250K (above T_N). At 230K the applied out-of-plane magnetic field switches the weak net moment with a coercive field of a 0.4 T and a saturation field of several Tesla. The relatively large coercive field suggests strong crystalline anisotropy. The ~8 mμ_B/Mn saturation moment is much smaller than the 2.7 μ_B/Mn moment that would arise from fully-aligned Mn spins in this structure. We further investigated temperature dependent neutron diffraction measurements on these high-quality Mn_3NiN thin films (fig. S8C and fig. S9). Interestingly, the Mn_3NiN (001) magnetic diffraction peak was observed below T_N, consistent with the onset of long-range AFM Γ_{4g} spin ordering. The magnetic peak intensity remains almost constant down to 2 K (fig. S9). Thus, we conclude the presence of robust Γ_{4g} phase in these Mn_3NiN films.

Transport characterization of Mn_3NiN thin films

In a conventional Fermi liquid, the low-temperature resistivity follows a T^2 dependence due to dominant electron-electron scattering. In our Mn_3NiN films, however, the best fit to the low-temperature resistivity yields a power law close to $T^{5/3}$ (≈1.7), slightly reduced from the ideal T^2 behavior. Such an exponent is commonly associated with enhanced spin-fluctuation scattering in three-dimensional itinerant magnetic systems and represents a well-known deviation from simple Fermi-liquid behavior near magnetic instabilities.

Within spin-fluctuation theory for itinerant magnets, critical magnetic fluctuations modify the quasiparticle scattering rate and can produce a $T^{5/3}$ dependence of resistivity in 3D systems. In our case, the noncollinear

Γ_{4g} magnetic ordering and the proximity to magnetic fluctuations provide a natural source of such additional scattering. Therefore, the observed exponent suggests that, while Mn_3NiN remains a good metal, its low-temperature transport is influenced by magnetic (spin) fluctuations rather than purely conventional electron-electron interactions.

Importantly, the exponent being close to 2 still indicates coherent metallic transport with well-defined quasiparticles, consistent with the sizable anomalous Hall response and Kerr signal observed in our measurements. In contrast to many collinear antiferromagnets that are insulating or semimetallic, Mn_3NiN exhibits robust metallicity with fluctuation-modified scattering.

Sagnac MOKE characterization of Mn_3NiN thin films

In noncollinear antiferromagnets, magneto-optical Kerr effect (MOKE) does not require a net magnetization. As established theoretically and experimentally (e.g., *(11, 53)* symmetry breaking combined with spin-orbit coupling can generate both an anomalous Hall effect (AHE) and a finite Kerr response in materials with nearly zero net moment. These effects originate from the same symmetry-allowed Berry curvature in momentum space.

The intrinsic anomalous Hall conductivity (AHC) is a dc response obtained by integrating the Berry curvature over all occupied states up to the Fermi level. In contrast, MOKE is governed by the finite-frequency off-diagonal optical conductivity $\sigma_{xy}(\omega)$, which involves interband transitions between occupied and unoccupied states. Importantly, in the $\omega \rightarrow 0$ limit, $\sigma_{xy}(\omega)$ continuously approaches the intrinsic dc Hall conductivity. Thus, MOKE can be viewed as the finite-frequency extension of the intrinsic AHE. Although energy weighting differs, both responses are determined by the same Berry-curvature-induced breaking of time-reversal symmetry and crystal symmetries.

For Mn_3NiN, the Γ_{4g} noncollinear magnetic structure produces finite Berry curvature through spin-orbit coupling and magnetic symmetry breaking. This Berry curvature gives rise to the intrinsic AHE and, correspondingly, to a finite Kerr signal despite the almost absence of a net magnetization. Experimentally, we observe that the Kerr and Hall signals track the same magnetic domain configuration, demonstrating their common microscopic origin.

Spot size and spatial resolution: The Sagnac MOKE measurements were performed with a laser spot size of approximately ~1.5 µm, which defines the lateral spatial resolution of the technique. The pixel size used during scanning (i.e., the step size between measurement points in Fig. 3) was approximately ~1 µm. The Kerr sensitivity of the interferometric Sagnac setup is on the order of ~10 nrad, enabling detection of very small magneto-optical signals.

Theory calculation of Mn_3NiN thin films

The first nearest neighbor Heisenberg exchange energy J_1 is calculated via DFT by comparing the energy of the Γ_{4g} (fig. S23A) and ferromagnetic (fig. S23C) phases, as follows:

$$E^{\Gamma 4g} = E_0 + 6J_1 - 18J_2 \tag{S1}$$

$$E^{\mathrm{FM}} = E_0 - 12J_1 - 18J_2 \tag{S2}$$

$$J_1 = \frac{E^{\Gamma 4g} - E^{\mathrm{FM}}}{18} = -23.85\ meV \tag{S3}$$

The second nearest neighbor exchange energy J_2 is obtained by varying to fit experimentally measured T_N (fig. S23D).

The single ion magnetic anisotropy form respects the Mn atom 4/mmm site symmetry of the Wykoff position 3c in its space group Pm-3m. The positive (negative) sign of the anisotropy constant k results in the noncollinear antiferromagnetic phase $\Gamma_{4g(5g)}$, while increasing the magnitude of k monotonically increases the net moment. It is calculated via DFT by comparing the energy of the Γ_{4g} (fig. S23A) and Γ_{5g} (fig. S23B) phases, as follows:

$$E^{\Gamma 4g} = E'_0 - 3k\left(\frac{2}{\sqrt{6}}\right)^2 \quad (S4)$$

$$E^{\Gamma 5g} = E'_0 - 3k\,(0)^2 \quad (S5)$$

$$k = \frac{E^{\Gamma 4g} - E^{\Gamma 5g}}{2} = -0.133\,meV \quad (S6)$$

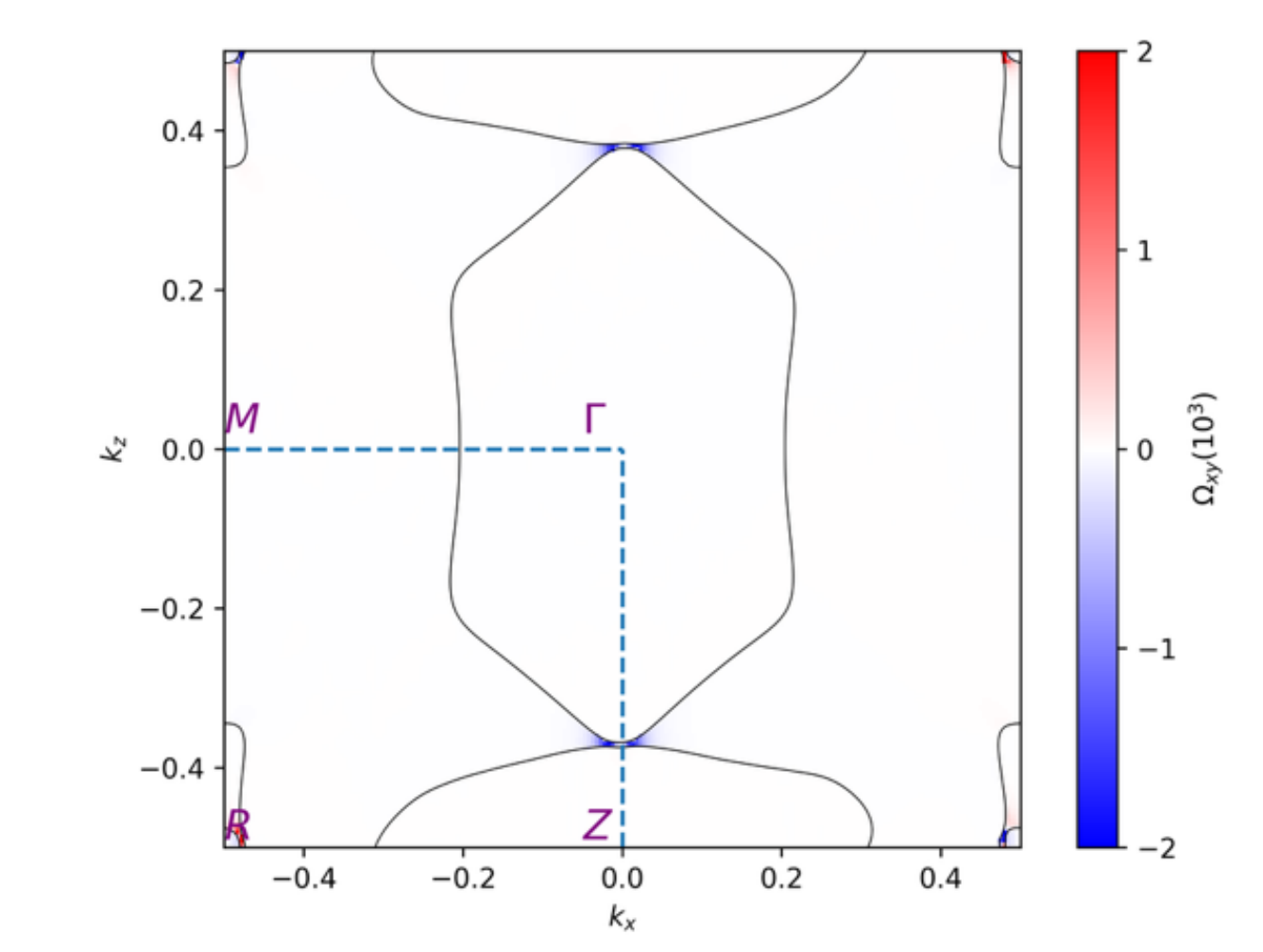

Fig. S1. First-principles calculation of the Berry curvature of Mn_3NiN. The calculated color map of the Berry curvature in the (110) plane for the Γ_{4g} phase of Mn_3NiN.

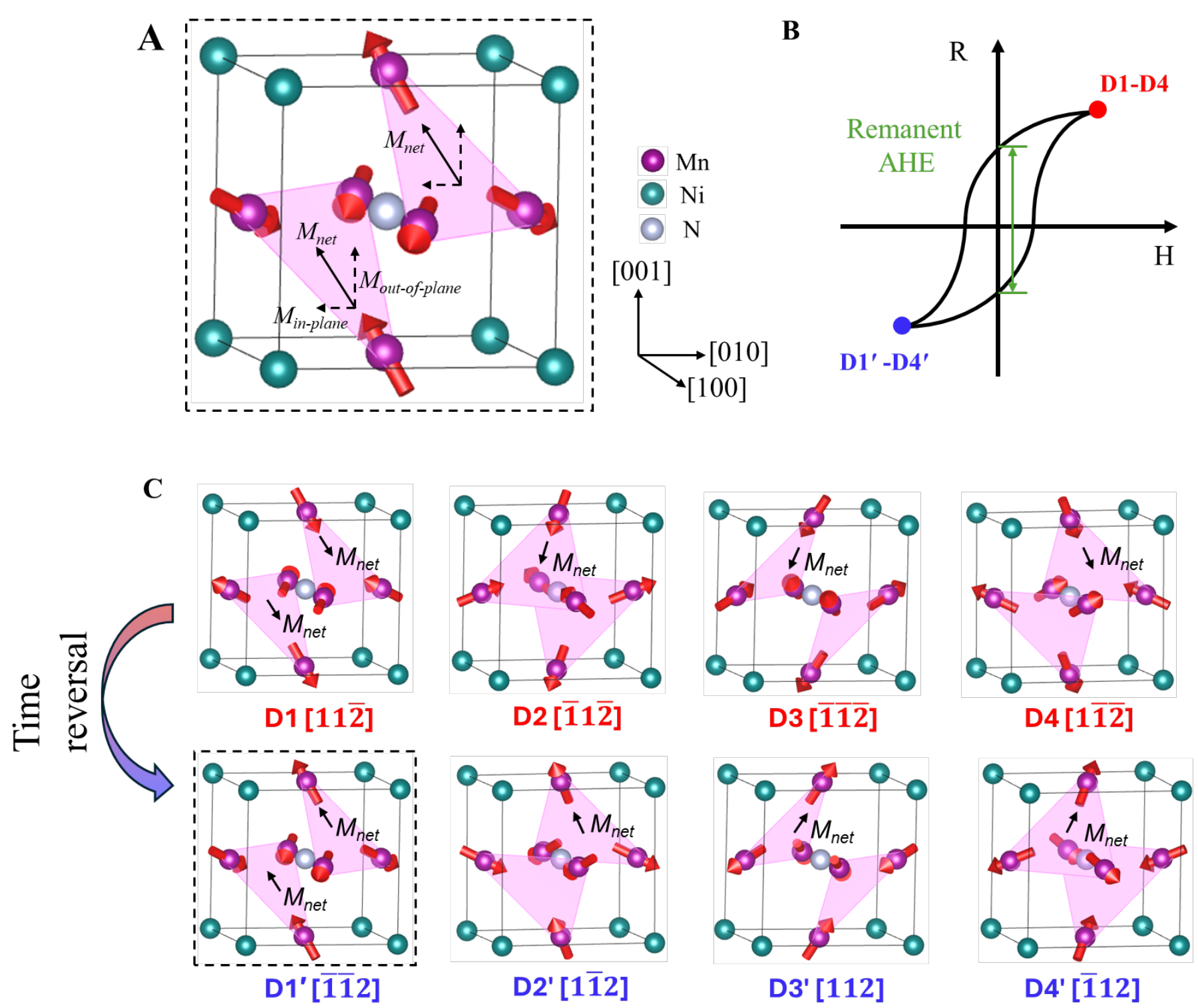

Fig. S2. Schematic illustration of Mn_3NiN. (A) antiperovskite Mn_3NiN with net moment induced from a distortion in Γ_{4g} spin structure, (B) domain contribution on Berry curvature driven anomalous Hall effect in Mn_3NiN, and (C) all 8 possible antiferromagnetic domain configurations of Mn_3NiN with their moment directions.

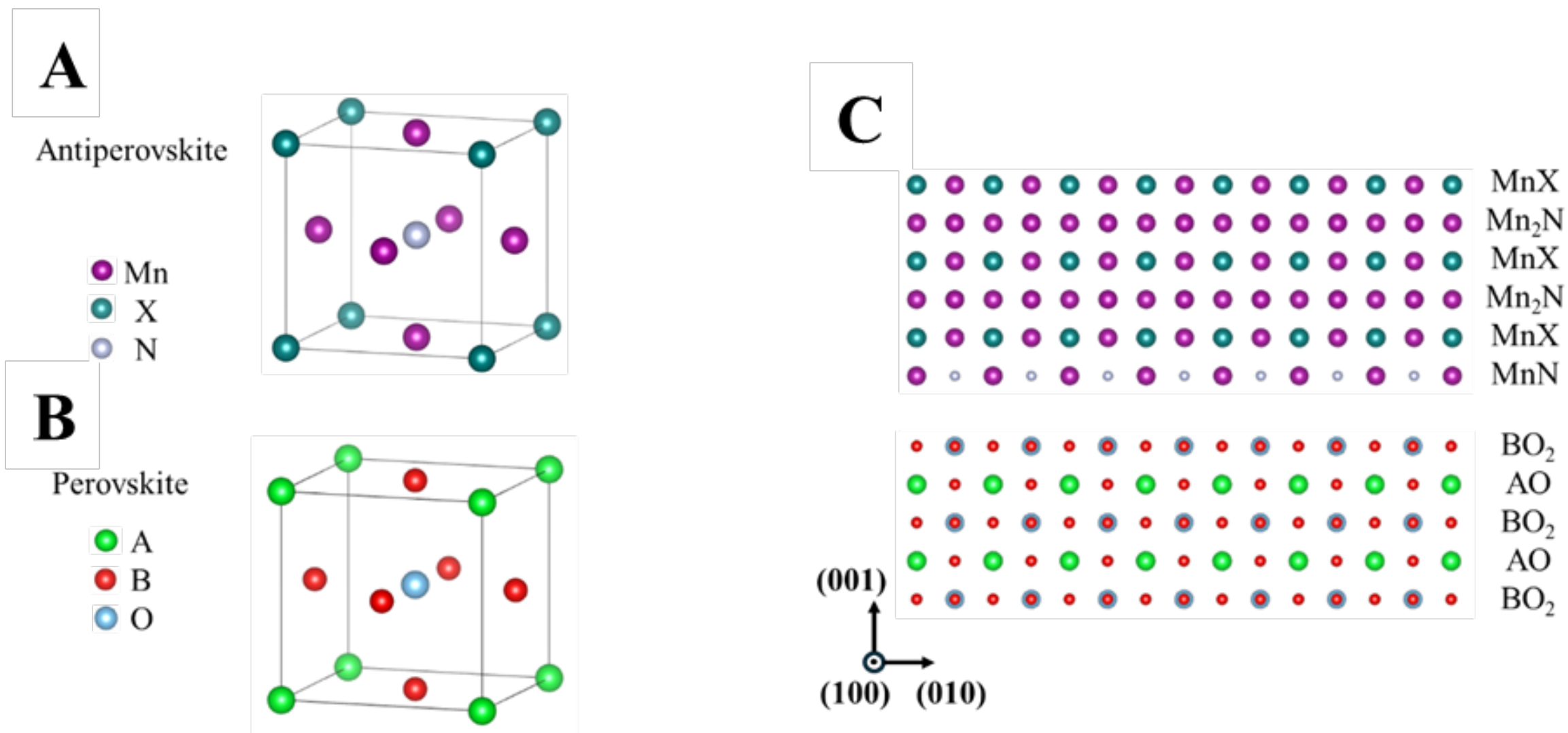

Fig. S3. Antiperovskite and perovskite heterostructure. Schematic illustrations of **(A)** antiperovskite nitride unit cell, **(B)** perovskite oxide unit cell. **(C)** Stacking of antiperovskite nitride on perovskite oxide in thin film heterostructure.

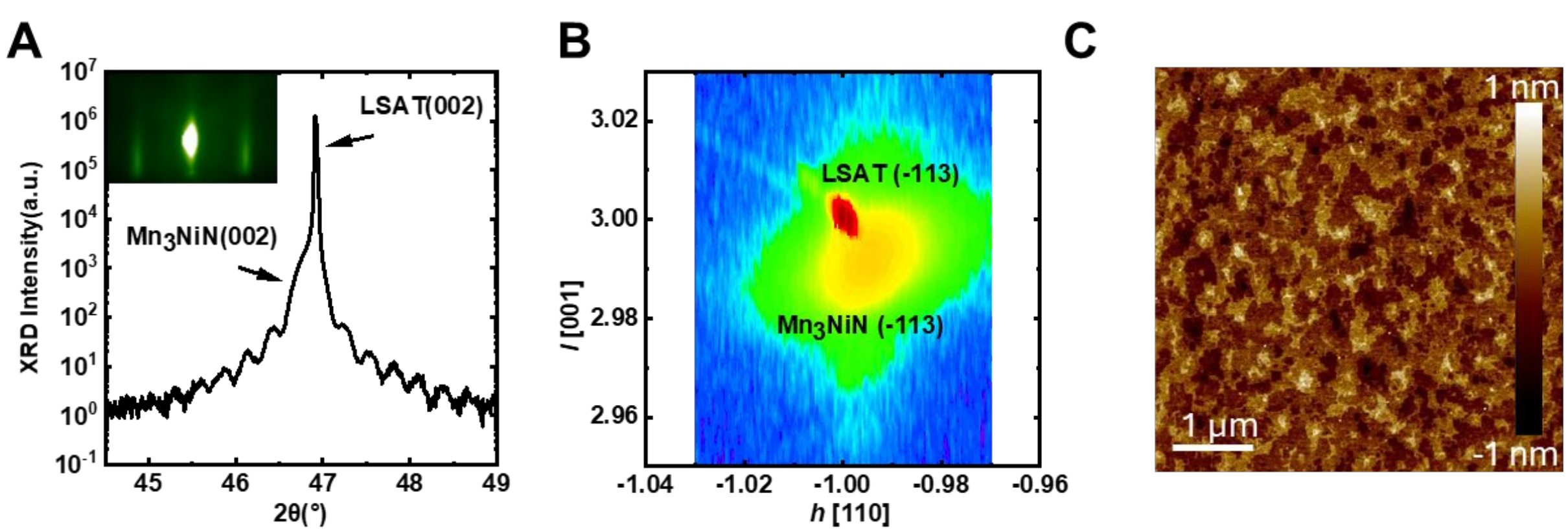

Fig. S4. Structural and topographical characterization. (A) θ-2θ spectrum around (002) peak of the LSAT substrate and the Mn_3NiN film with the streaky RHEED pattern as inset, demonstrating that the high-quality growth of film with (001)-oriented and single phase. **(B)** X-ray reciprocal space mapping shows tetragonal distortion of Mn_3NiN thin film. **(C)** atomic force imaging of Mn_3NiN thin film, with the atomic level smoothness.

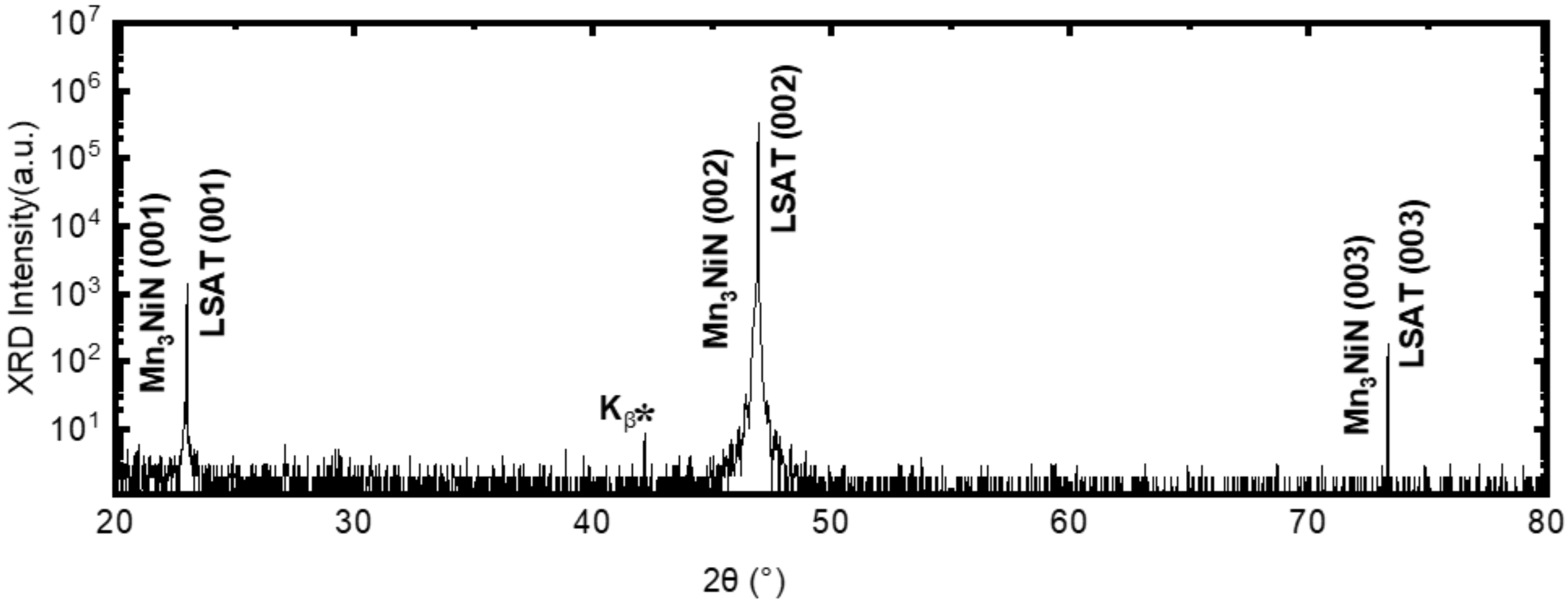

Fig. S5. Structural characterization of Mn_3NiN thin films. Wide-angle θ-2θ spectrum only shows the (00l) reflections of the LSAT substrate and the Mn_3NiN film, demonstrating that the film is (001)-oriented and single phase.

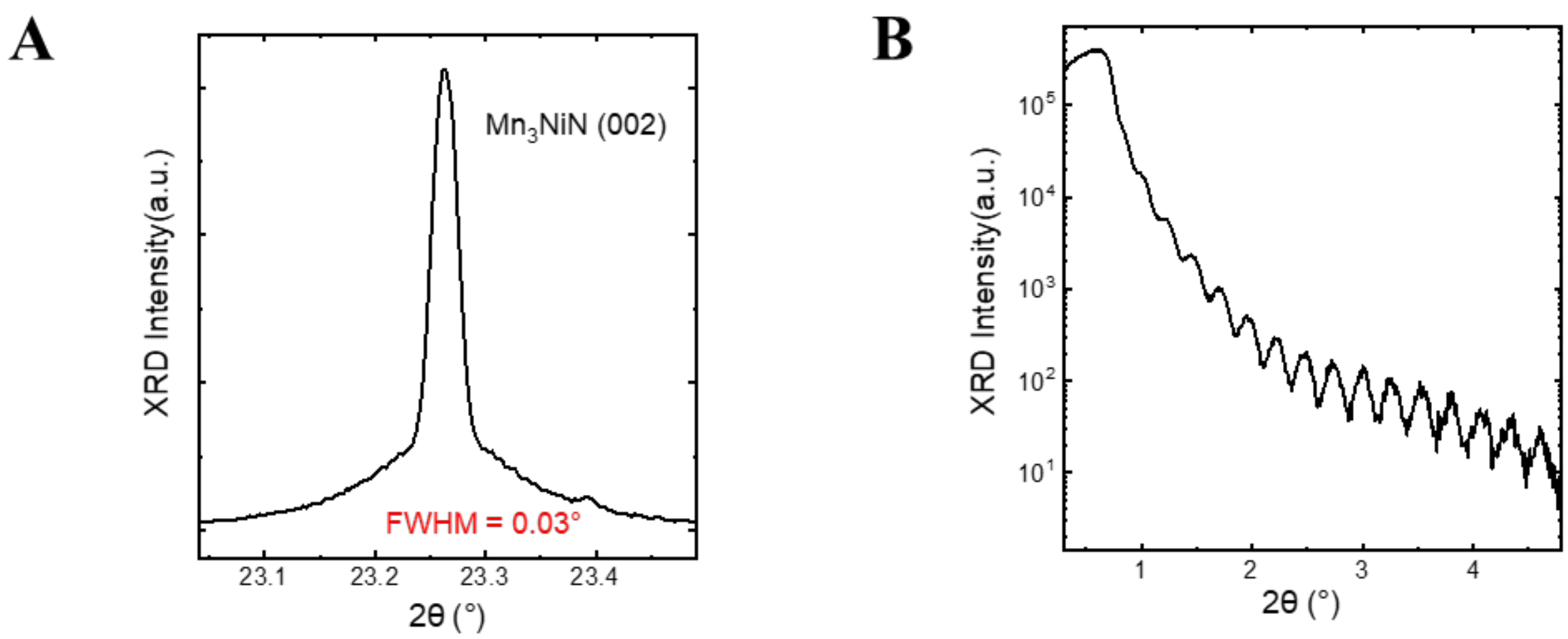

Fig. S6. X-ray reflectivity and rocking curve analyses. (A) X-ray reflectivity spectrum showing a pristine interface with clear fringes. **(B)** Rocking curve of (002) Mn_3NiN peak indicating high crystallinity with FWHM of 0.029º.

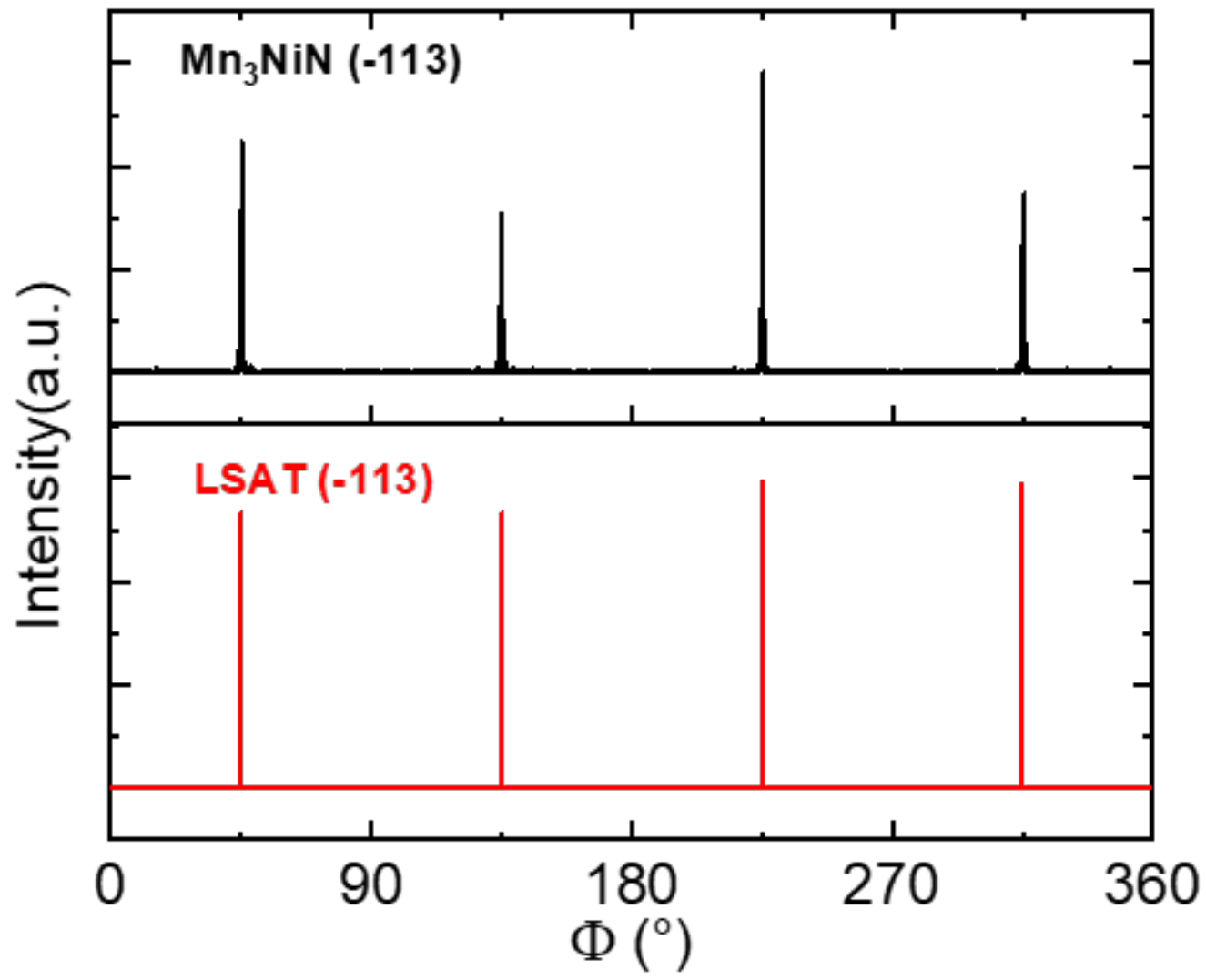

Fig. S7. Phi-scans. Phi-scans at asymmetric (-113) peak of Mn_3NiN and LSAT indicates in-plane cube-on-cube epitaxial relationship.

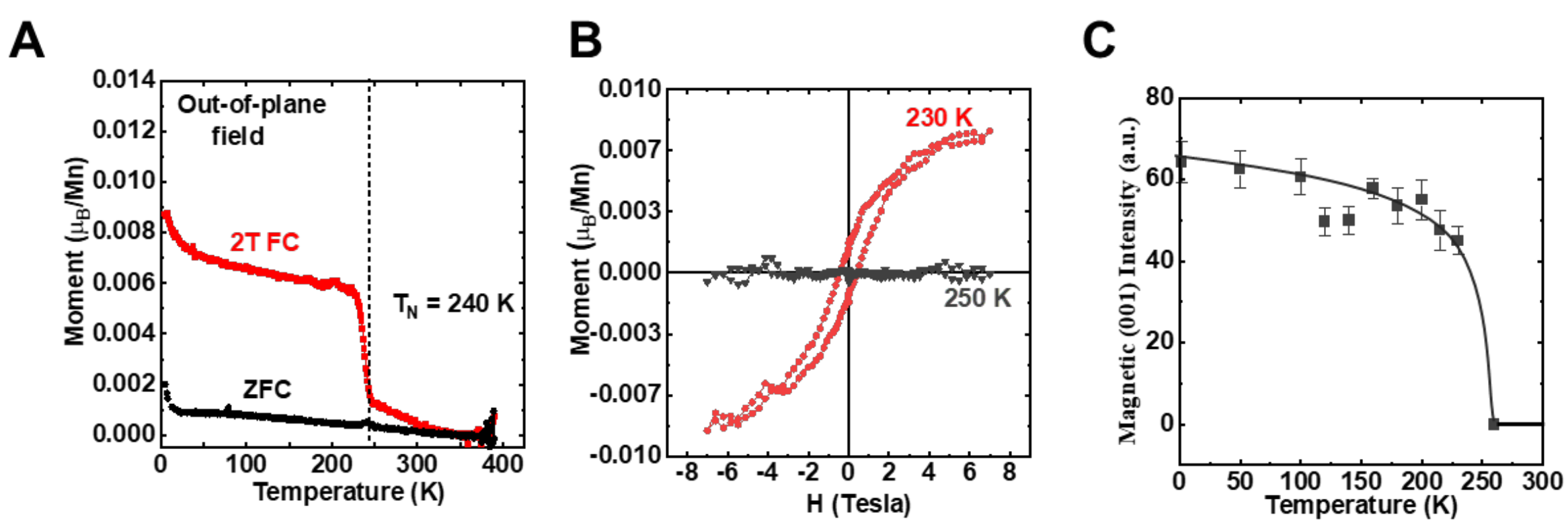

Fig. S8. Magnetic characterization of Mn_3NiN thin films. (A) Out-of-plane net magnetization vs temperature curves for 2 T field cooled (red) and zero-field-cooled (black), while measuring during warming in 0.5 T. Distinct Néel transitions are visible around 240 K. **(B)** Net magnetization vs out-of-plane magnetic field showing clear weak net moment below T_N. **(C)** Neutron diffraction measurement on a 250nm Mn_3NiN films, temperature dependent neutron diffraction intensity indicate the Γ^{4g} phase in Mn_3NiN.

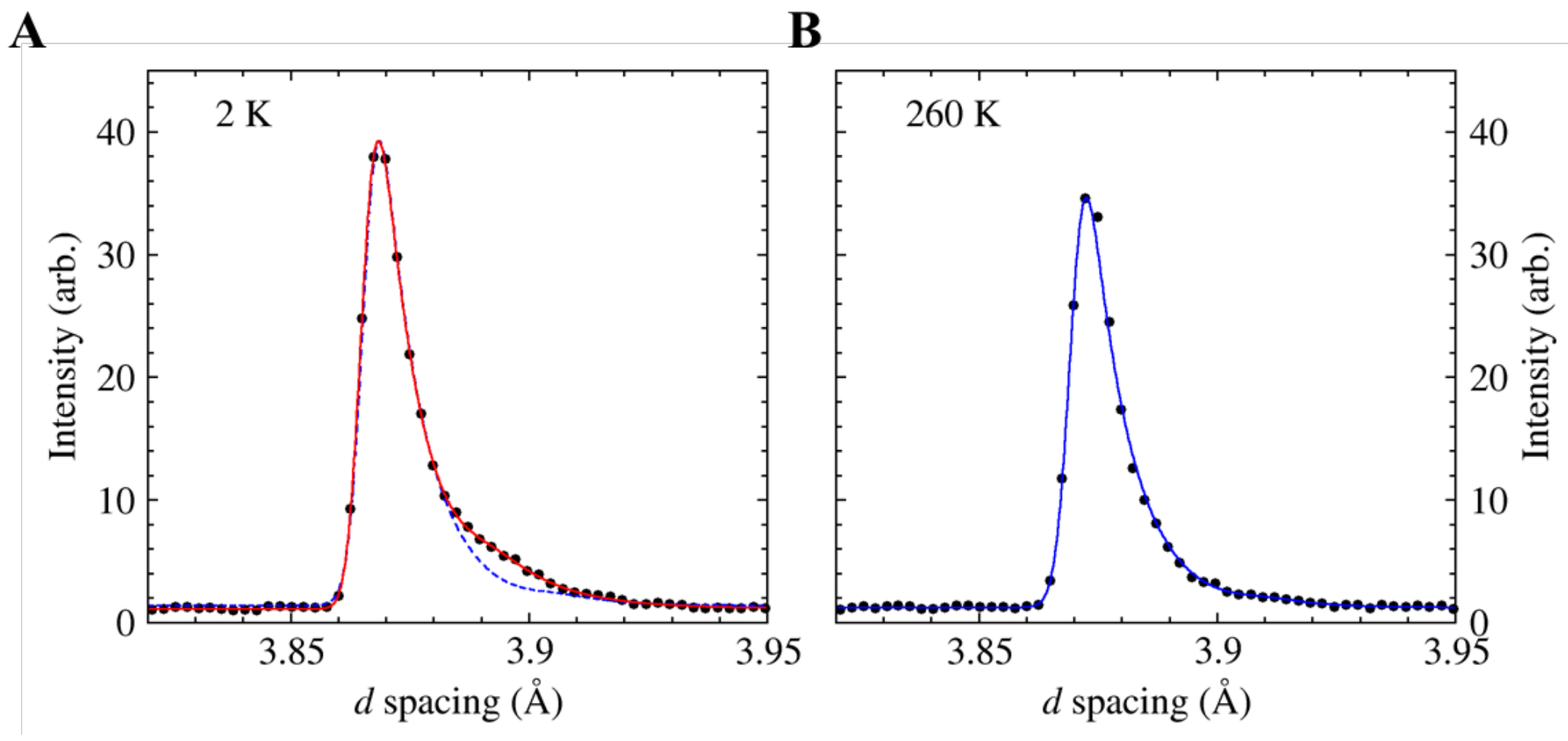

Fig. S9. Temperature dependent neutron diffraction data. A single 250 nm Mn_3NiN thin film sample was mounted within a ^{4}He cryostat and oriented for scattering in the HHL reciprocal space plane in a geometry optimized for peak flux and resolution at the (001) Bragg reflection. Neutron diffraction intensity (black points) at the (001) reciprocal space position defined with respect to the antiperovskite crystal structure, measured at **(A)** 2 K and **(B)** 260 K. The 2 K intensity includes contributions from both nuclear diffraction of the substrate and magnetic diffraction of the film, while the 260 K data include only diffraction from the substrate. The 2 K data have been fit using two back-to-back exponential peak profiles (red line), and the 260 K data have been fit with a single back-to-back exponential peak profile (blue line). The single 260 K peak profile has been overlaid in pane **(A)**, shifted and scaled to the more intense substrate peak (blue dashed line), revealing the weak magnetic intensity from the film at longer *d*-spacing.

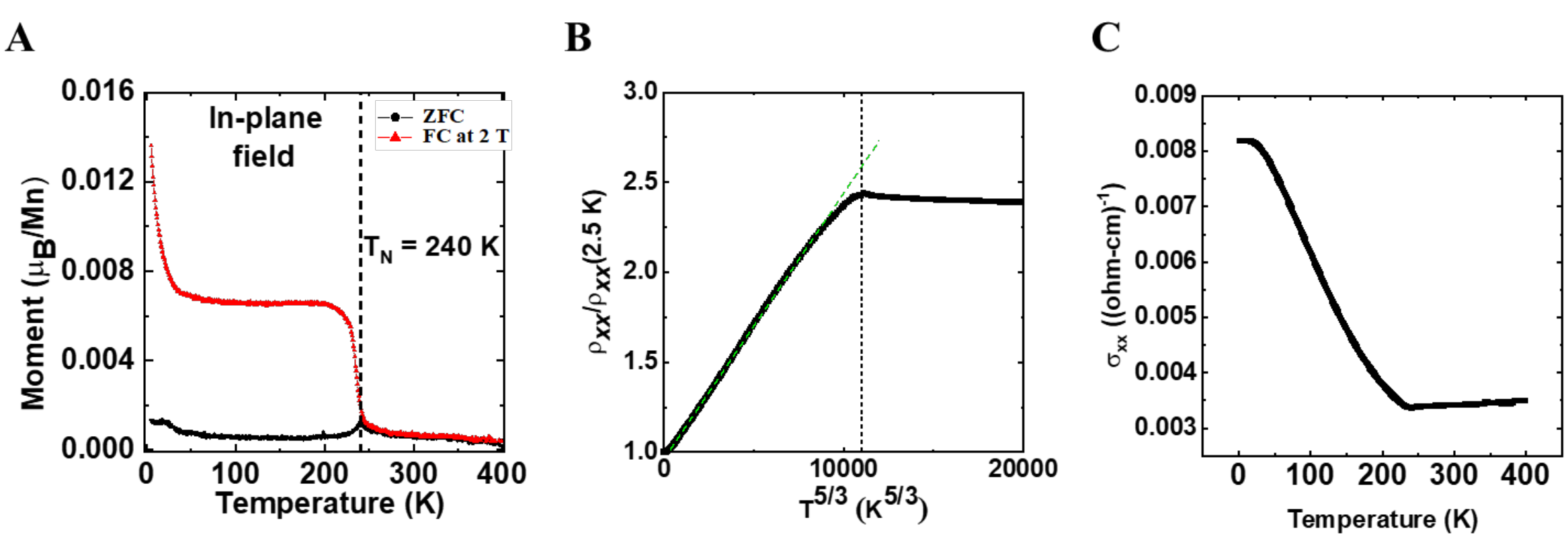

Fig. S10. In-plane magnetization data and residual resistivity plot. (A) In-plane net magnetization vs temperature curves for field cooled (red) and zero-field-cooled (black). **(B)** Temperature dependent variation of sheet resistivity and **(C)** Temperature dependent variation of sheet conductivity showing excellent metallic behavior below T_N as of 240K.

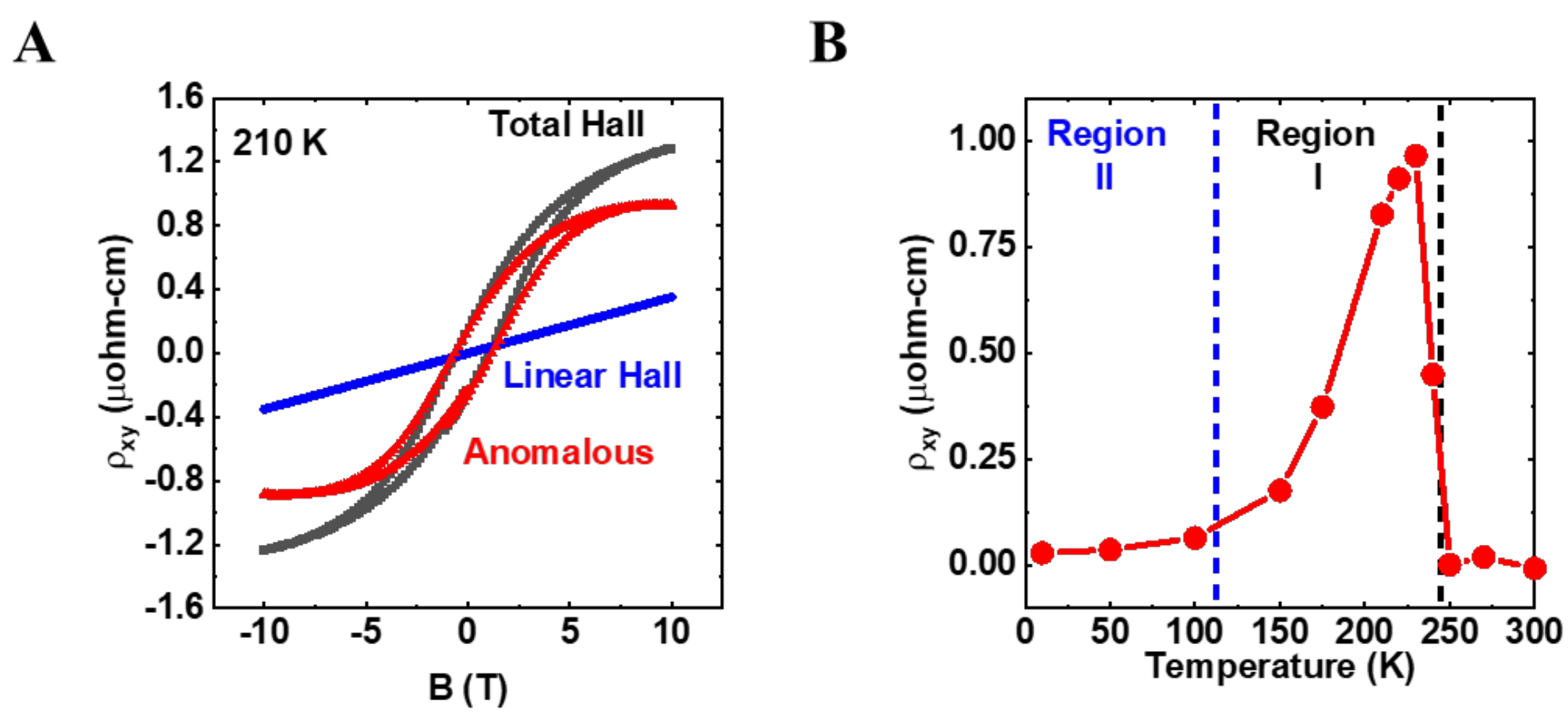

Fig. S11. Magneto transport measurements in a Hall-bar device. (A) Hall resistivity vs out of plane magnetic field at 210 K, which also shows both normal linear part and anomalous part separated from the total. **(B)** Temperature-dependent anomalous Hall resistivity.

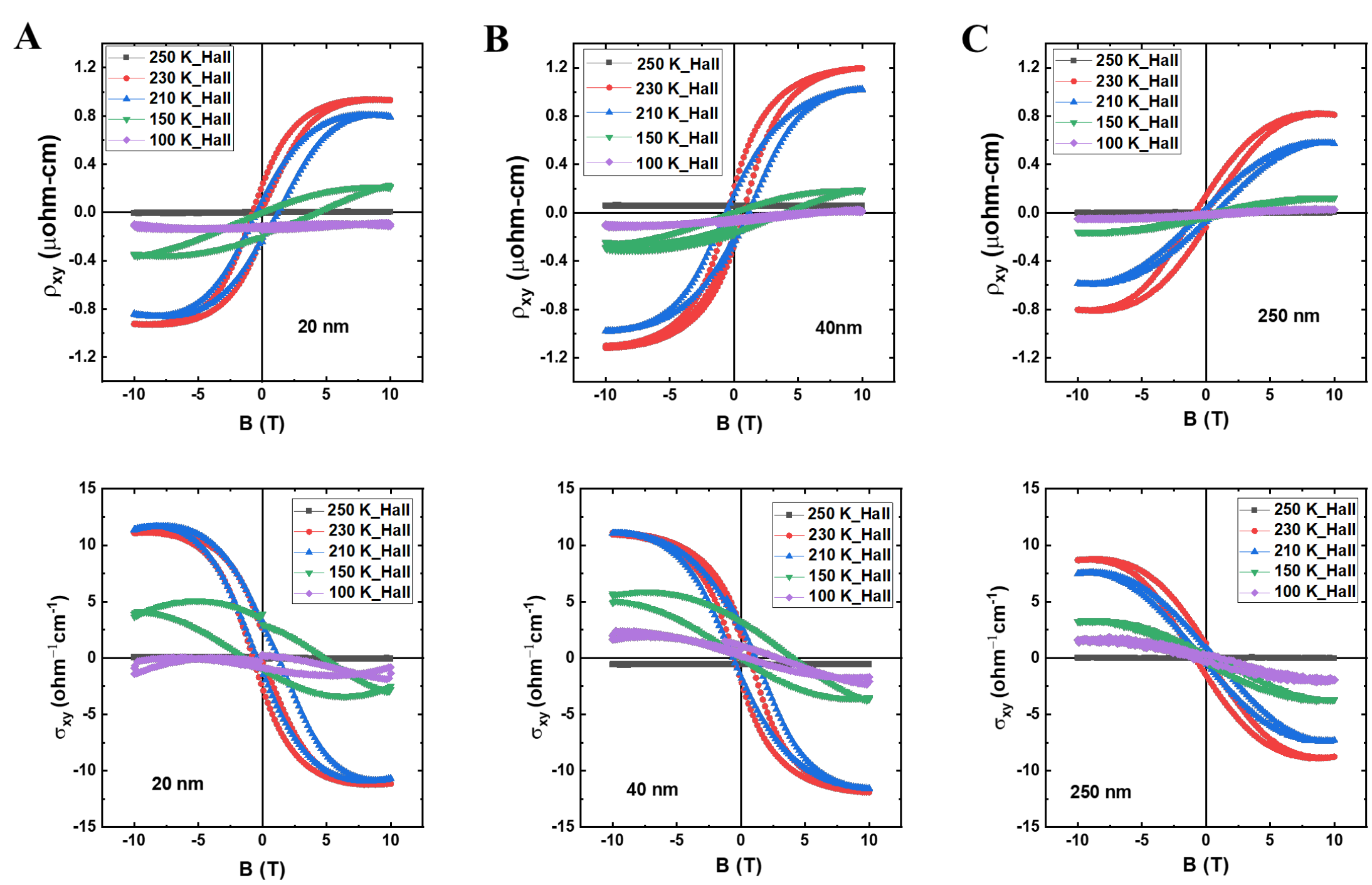

Fig. S12. Thickness dependent AHE in Mn_3NiN. Temperature dependent of anomalous Hall resistivity and conductivity of **(A)** 20nm, **(B)** 40nm and **(C)** 250nm Mn_3NiN single-crystal thin-films

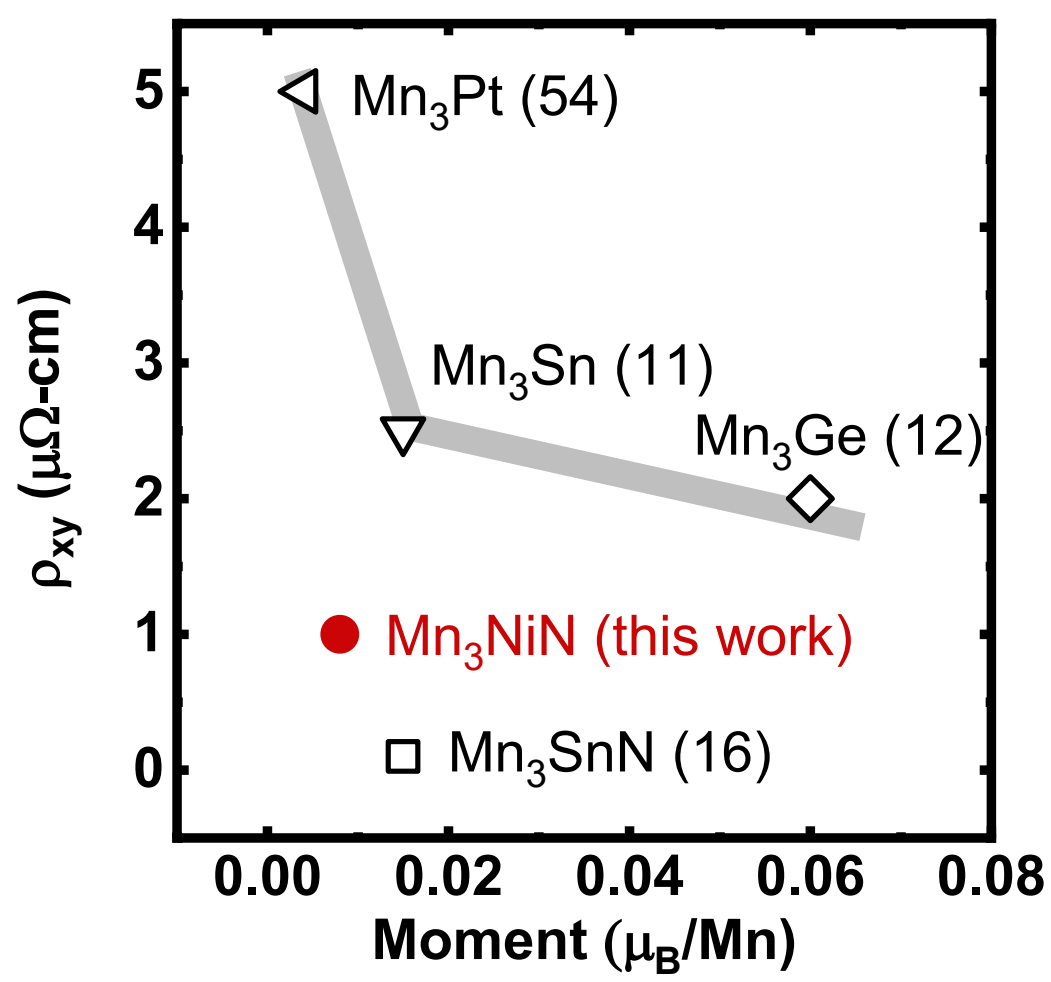

Fig. S13. Comparison of AHE with other non-collinear antiferromagnetic materials Comparison of AHE and net moment of Mn_3NiN in this work with other non-collinear antiferromagnetic materials with the reference (*11*, *12*, *16*, *54*).

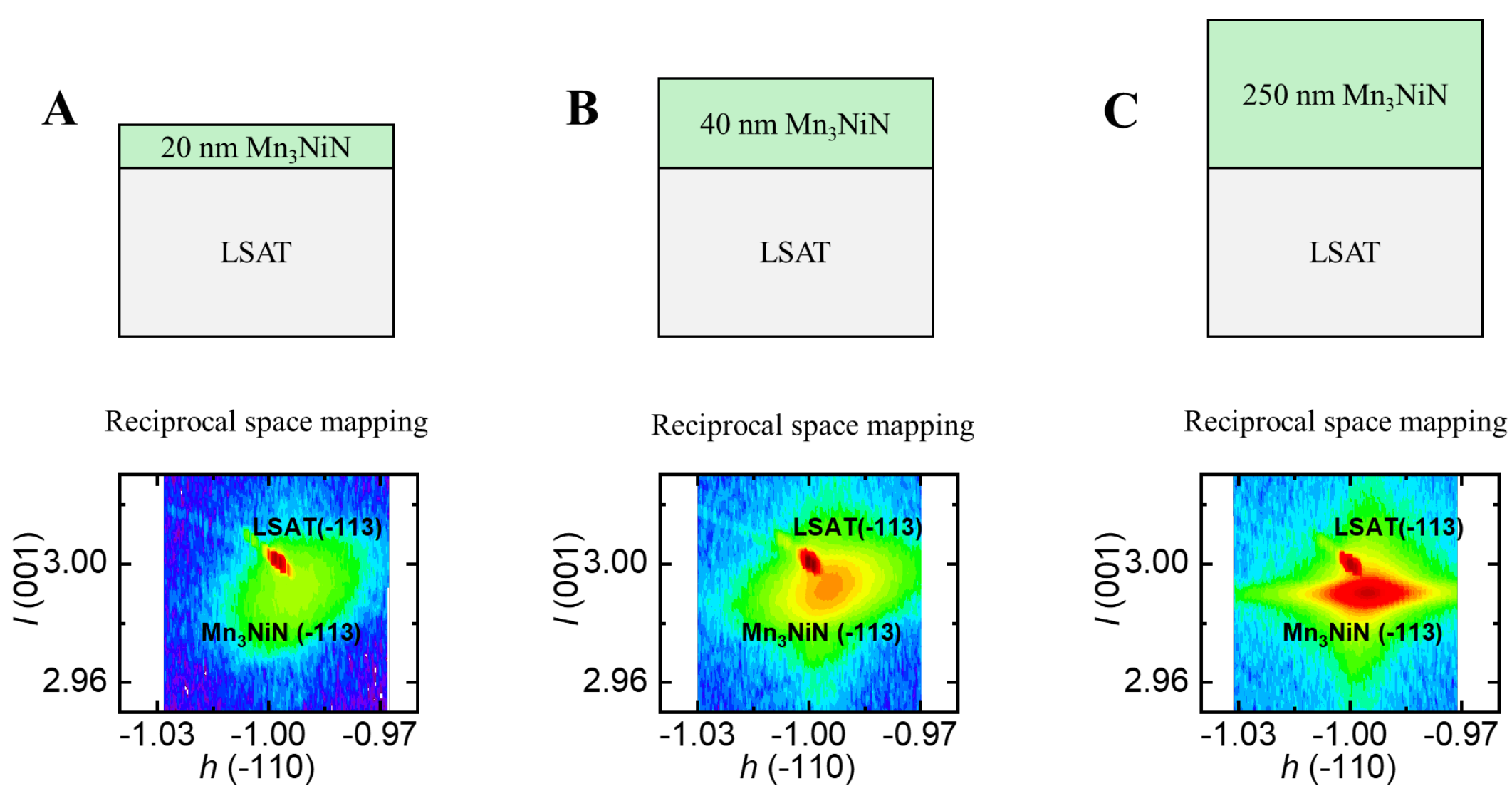

Fig. S14. Reciprocal space mapping of Mn_3NiN with (A) 20nm, (B) 40nm, and (C) 250nm. The reciprocal space mappings of varies thickness indicating the consistency of high crystallinity of Mn_3NiN epitaxial films.

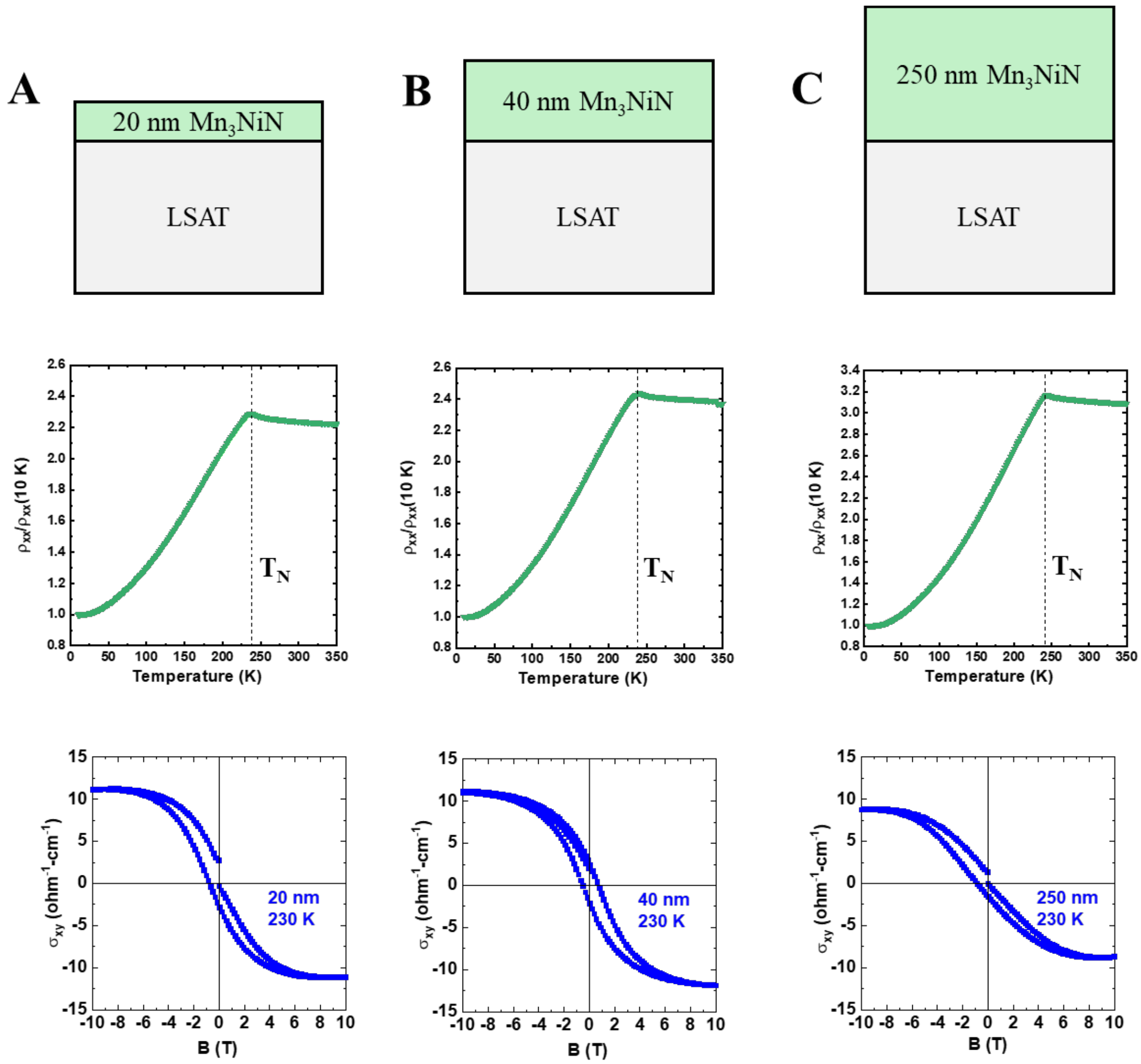

Fig. S15. Transport characterizations of Mn_3NiN with (A) 20nm, **(B)** 40nm, and **(C)** 250nm. The kinks at the ρ_{xx} vs T indicate the T_N of each thickness of Mn_3NiN films to be all ~240K. The σ_{xy} vs H indicate the observation of large anomalous Hall effect just below T_N.

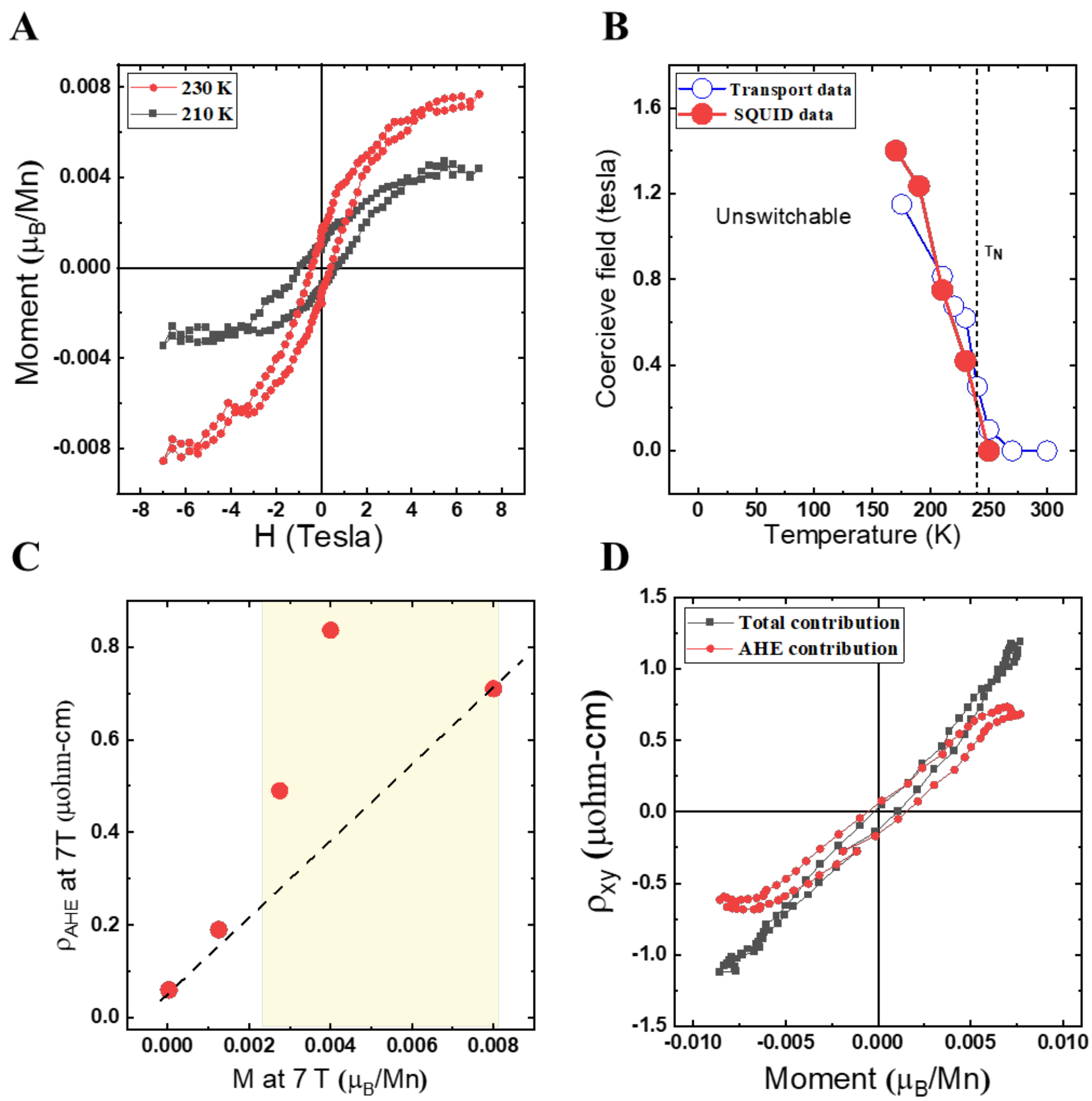

Fig. S16. Temperature dependent variations of coercive field and remanent moment. **(A)** Isothermal M vs H measurements for out-of-plane applied magnetic field for Mn_3NiN thin-films. **(B)** Variation of extracted coercive field with temperature. Increasing Coercive field below T_N, indicates enhanced magnetic domain anisotropy. **(C)** Variation of extracted ρAHE at 7 T with the corresponding magnetization obtained at various temperatures shows clear anomalous behavior. **(D)** Variation of ρxy at 230 K with the corresponding magnetization data, which shows deviation indicating different origin of AHE than simple magnetism.

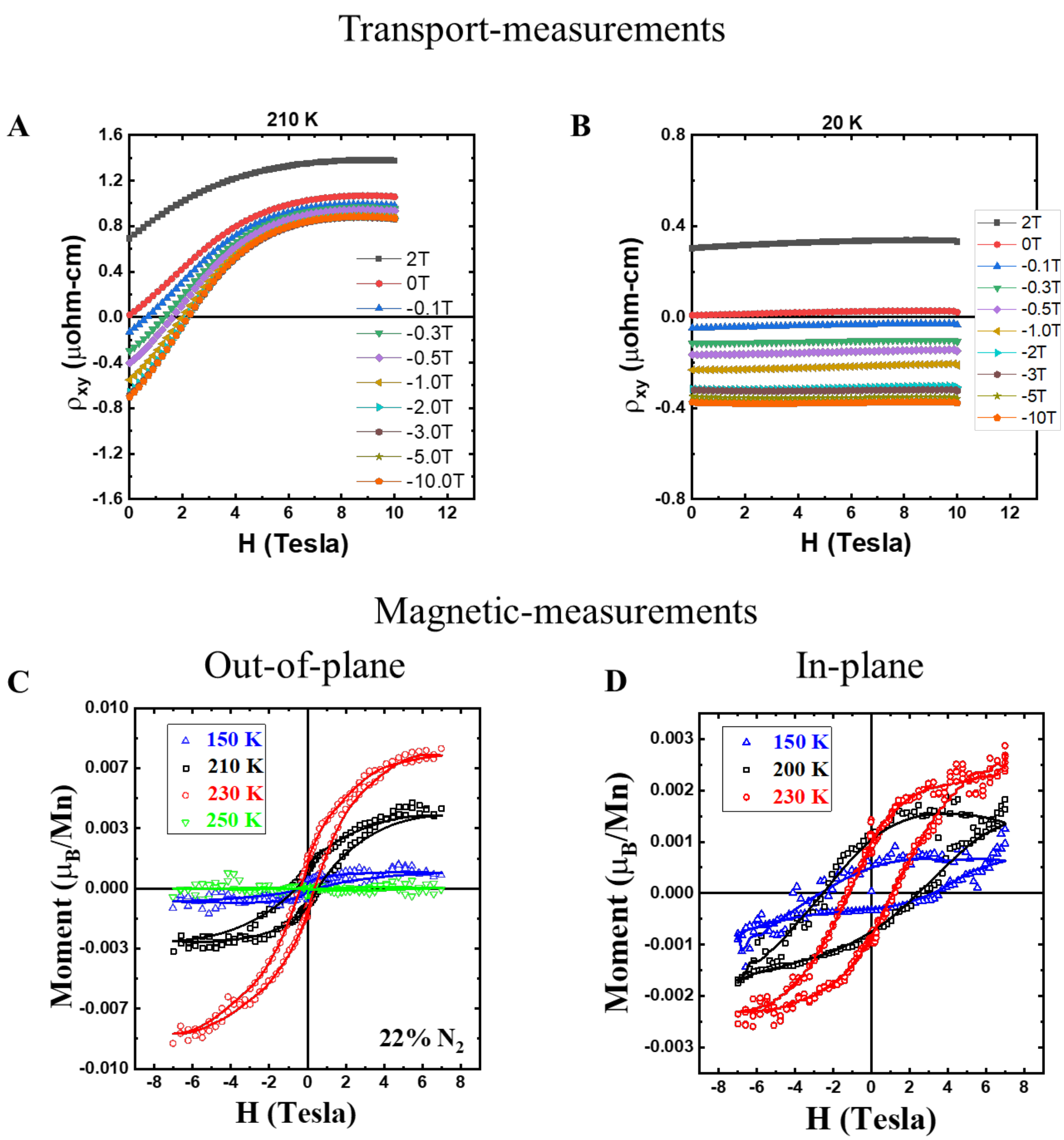

Fig. S17. Cooling filed dependent transport and Temperature dependent magnetic characterizations. Mn_3NiN was cooled with 2, 0, -0.1, -0.3, -0.5, -1, -2, -3, -5, and -10T, and then performed Hall vs H measurement at (A) 210K and (B) 20K. The variation of remanent Hall resistivity indicating the alignment antiferromagnetic domains through different field cooling. The lateral switchable and unswitchable anomalous Hall resistivity vs field at 210K and 20K are consistent with the idea of domain frozen in this manuscript. The magnetic measurement (C) and the coercive field of both magnetic and transport (D) indicated the gradual frozen of antiferromagnetic domains.

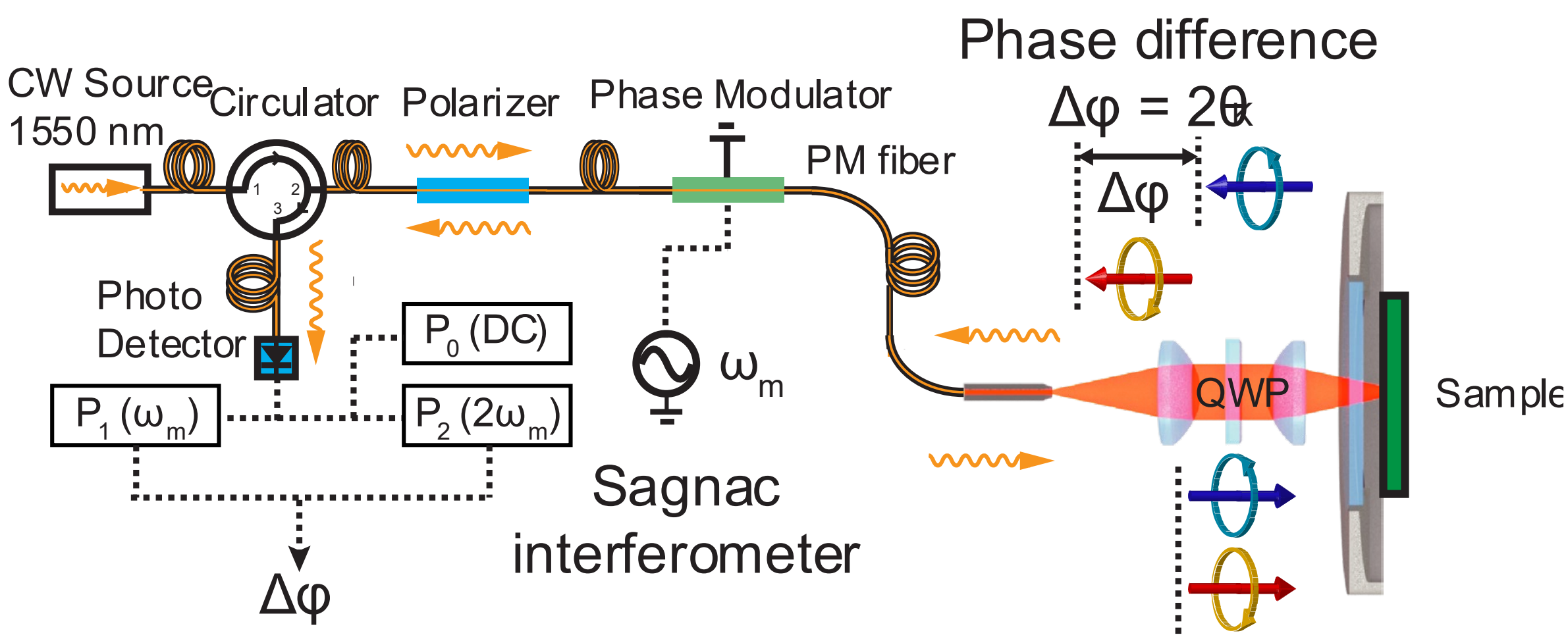

Fig. S18. Schematic drawing of the basic setup of Sagnac MOKE interferometry. MOKE signal θ_K is measured by a Sagnac zero-area-loop fiber-optic interferometer during varying temperature and sweeping magnetic field.

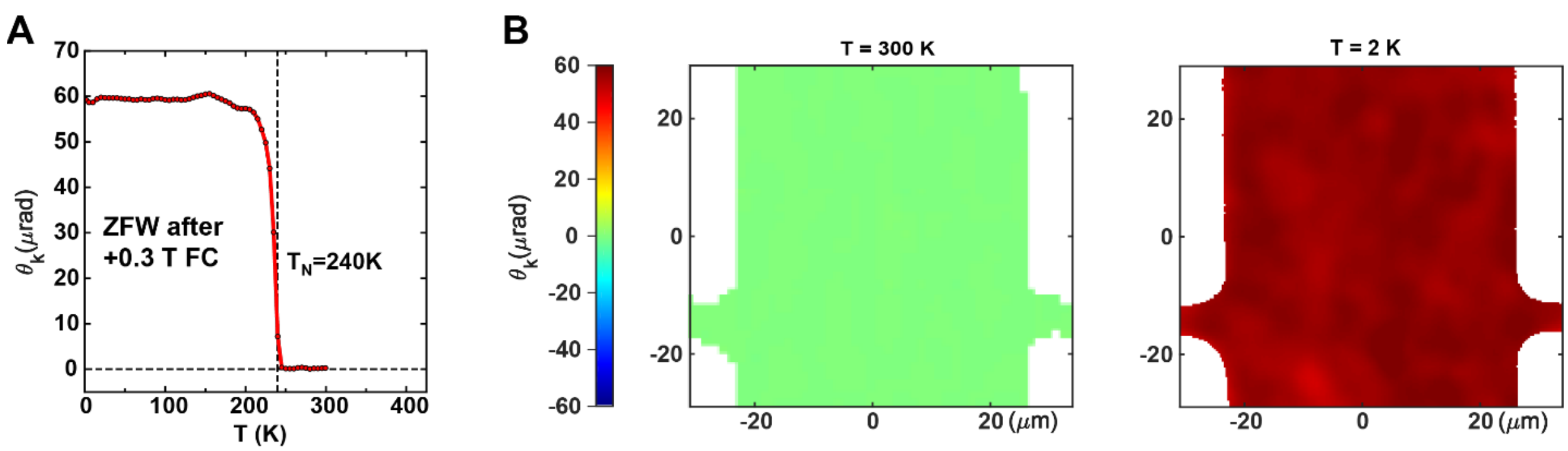

Fig. S19. Temperature dependent Sagnac MOKE characterization of Mn_3NiN thin films. (A) Temperature dependence of the Kerr rotation θ_k measured during zero field warmup after +0.3 T field cooling (FC), showing an onset of $\theta_k \approx 60\ \mu rad$ below the Néel temperature $T_N \approx 240$ K. **(B)** θ_k imaging at 300 K and 2 K, before and after warming in zero field following a +0.3 T FC, demonstrating the emergence of a strong Kerr signal below T_N.

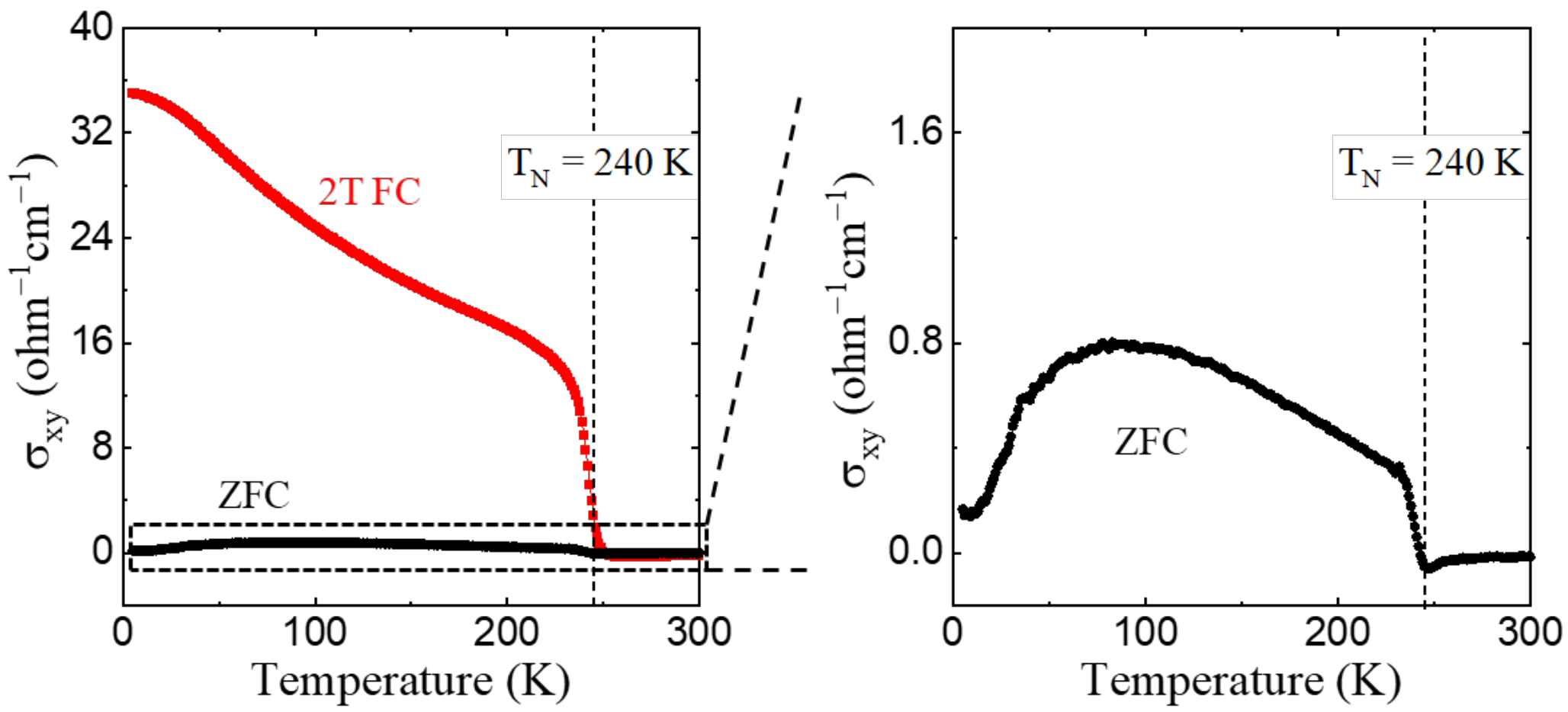

Fig. S20. Remanent anomalous Hall effect in Mn_3NiN film. Temperature dependence of remanent σ_{xy} during zero field warmup after 2 T field cooled (red) and zero-field-cooled (black). Distinct remanent anomalous Hall σ_{xy} are visible under 2T field cooling, and weak remanent σ_{xy} under zero field cooling indicates the nearly average domain formation during zero field cooling.

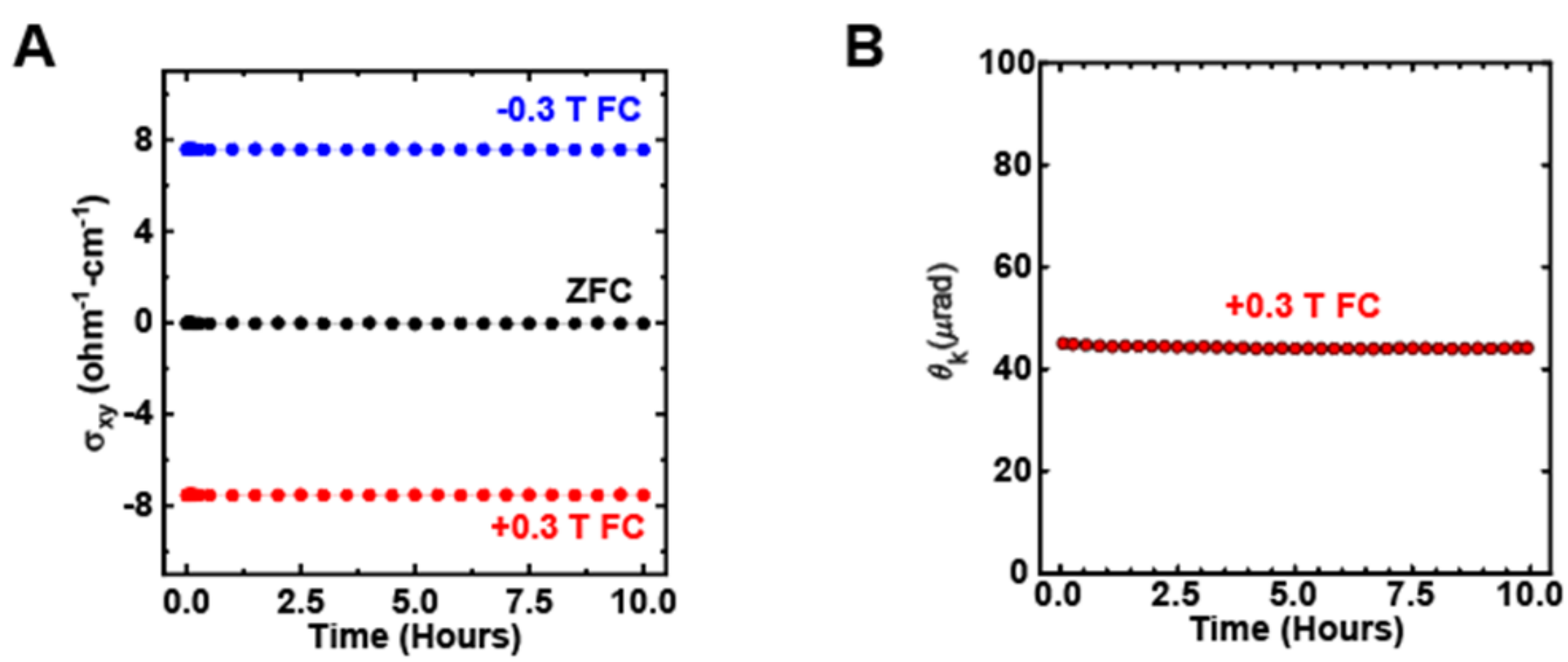

Fig. S21. Time dependent AHE and Kerr signal. Time dependent **(A)** anomalous Hall conductivity and **(B)** Kerr signal under +/- 0.3T and ZFC, showing the robust stability with the potential for permanent memory.

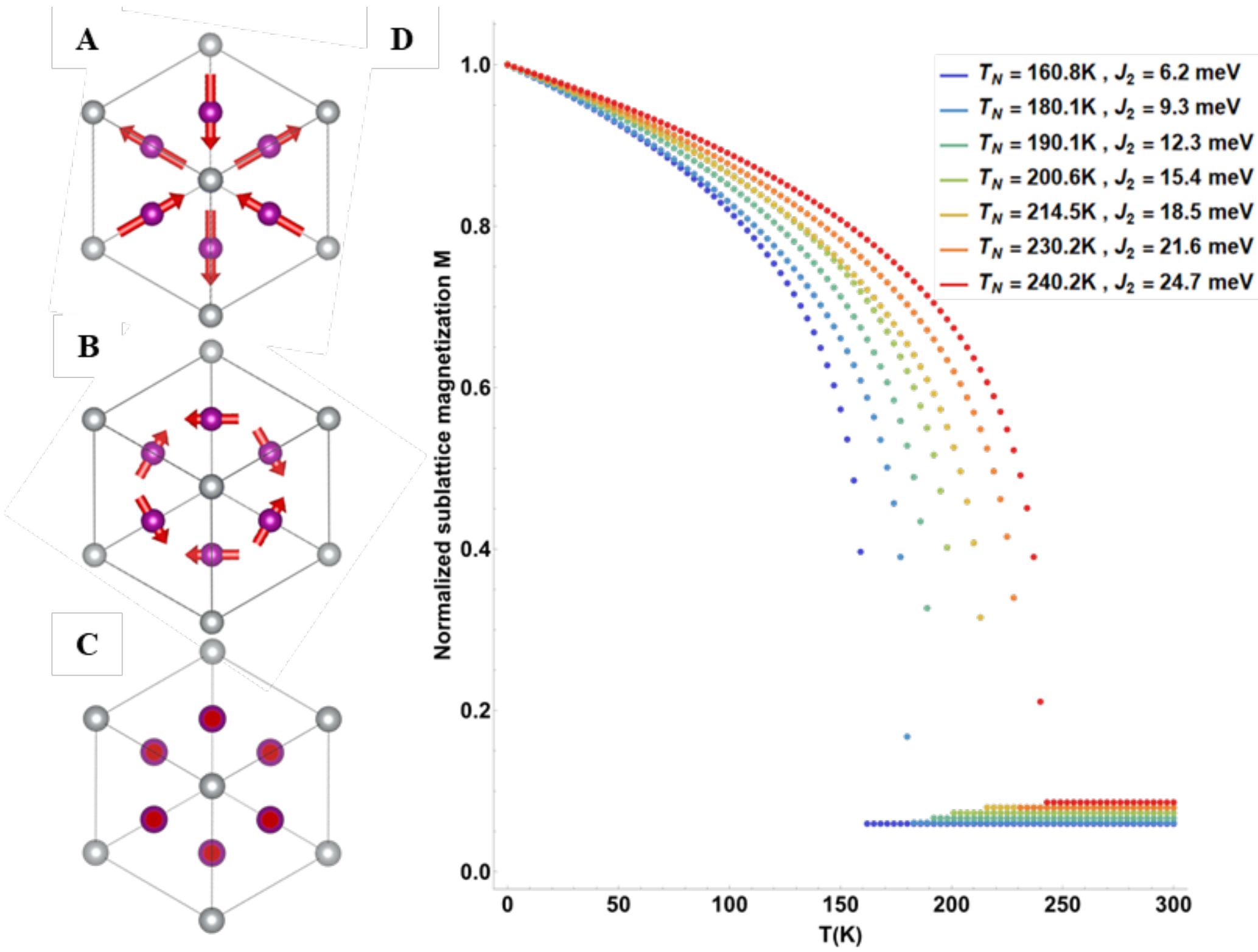

Fig. S22. Results of atomistic spin model simulations. Schematics of the Mn_3NiN magnetic unit cell projected to the (111) plane for noncollinear antiferromagnetic **(A)** Γ_{4g} and **(B)** Γ_{5g} phases, and **(C)** ferromagnetic phase. **(D)** Néel temperature T_N simulations for different second nearest neighbor exchange energy J_2.

Table S1: *Details of structural and physical properties parameters of Mn_3NiN thin films with varying thickness and their comparison. Bulk lattice parameters of Mn3NiN and Mn3GaN are 3.880Å and 3.898Å respectively [cite the references]. Lattice parameter of LSAT is 3.868 Å.*

Samples	In-plane lattice parameter (*a*=*b*)	Out-of-plane lattice parameter (*c*)	Néel Temperature (T_N)	RRR= ρ_{temp}/ρ (10K)	Peak σ_{xy} value at 230K ($\Omega^{-1}cm^{-1}$)	Reference
20 nm Mn_3NiN/LSAT	3.8873 Å	3.8834 Å	~240 K	2.3	11.1	This work
40 nm Mn_3NiN/LSAT	3.8994 Å	3.8807 Å	~240 K	2.43	11.3	This work
250 nm Mn_3NiN/LSAT	3.9029 Å	3.8806 Å	~240 K	3.18	8.7	This work
60 Mn_3GaN/LSAT	3.90 Å	3.92 Å	~350 K		0	(*15*) *Sci. Adv.*, **6**, eaba4017 (2020).
50 nm Mn_3NiN/LSAT	3.8787(3) Å	3.868 Å	250 K	1.49	~22	(*17*) *Phys. Rev. Materials* **3**, 094409 (2019)
100 nm Mn_3NiN/LSAT	3.876(1) Å	3.868 Å	~240 K		~12	(*17*) *Phys. Rev. Materials* **3**, 094409 (2019)

REFERENCES

1. S. D. Bader, S. S. P. Parkin, Spintronics. *Annu. Rev. Condens. Matter Phys.* **1**, 71–88 (2010).

2. I. Žutić, J. Fabian, S. Das Sarma, Spintronics: Fundamentals and applications. *Rev. Mod. Phys.* **76**, 323–410 (2004).

3. V. Baltz, A. Manchon, M. Tsoi, T. Moriyama, T. Ono, Y. Tserkovnyak, Antiferromagnetic spintronics. *Rev. Mod. Phys.* **90**, 15005 (2018).

4. R. O. Cherifi, V. Ivanovskaya, L. C. Phillips, A. Zobelli, I. C. Infante, E. Jacquet, V. Garcia, S. Fusil, P. R. Briddon, N. Guiblin, A. Mougin, A. A. Ünal, F. Kronast, S. Valencia, B. Dkhil, A. Barthélémy, M. Bibes, Electric-field control of magnetic order above room temperature. *Nat. Mater.* **13**, 345–351 (2014).

5. A. Bose, N. J. Schreiber, R. Jain, D.-F. Shao, H. P. Nair, J. Sun, X. S. Zhang, D. A. Muller, E. Y. Tsymbal, D. G. Schlom, D. C. Ralph, Tilted spin current generated by the collinear antiferromagnet ruthenium dioxide. *Nat. Electron.* **5**, 267–274 (2022).

6. P. Wadley, B. Howells, J. Železný, C. Andrews, V. Hills, R. P. Campion, V. Novák, K. Olejník, F. Maccherozzi, S. S. Dhesi, S. Y. Martin, T. Wagner, J. Wunderlich, F. Freimuth, Y. Mokrousov, J. Kuneš, J. S. Chauhan, M. J. Grzybowski, A. W. Rushforth, K. Edmond, B. L. Gallagher, T. Jungwirth, Spintronics: Electrical switching of an antiferromagnet. *Science* **351**, 587–590 (2016).

7. X. Chen, T. Higo, K. Tanaka, T. Nomoto, H. Tsai, H. Idzuchi, M. Shiga, S. Sakamoto, R. Ando, H. Kosaki, T. Matsuo, D. Nishio-Hamane, R. Arita, S. Miwa, S. Nakatsuji, Octupole-driven magnetoresistance in an antiferromagnetic tunnel junction. *Nature* **613**, 490–495 (2023).

8. P. Qin, H. Yan, X. Wang, H. Chen, Z. Meng, J. Dong, M. Zhu, J. Cai, Z. Feng, X. Zhou, L. Liu, T. Zhang, Z. Zeng, J. Zhang, C. Jiang, Z. Liu, Room-temperature magnetoresistance in an all-antiferromagnetic tunnel junction. *Nature* **613**, 485–489 (2023).

9. D. Boldrin, A. P. Mihai, B. Zou, J. Zemen, R. Thompson, E. Ware, B. V. Neamtu, L. Ghivelder, B. Esser, D. W. McComb, P. Petrov, L. F. Cohen, Giant piezomagnetism in Mn_3NiN. *ACS Appl. Mater. Interfaces* **10**, 18863–18868 (2018).

10. T. Nan, C. X. Quintela, J. Irwin, G. Gurung, D. F. Shao, J. Gibbons, N. Campbell, K. Song, S. Y. Choi, L. Guo, R. D. Johnson, P. Manuel, R. V. Chopdekar, I. Hallsteinsen, T. Tybell, P. J. Ryan, J. W. Kim, Y. Choi, P. G. Radaelli, D. C. Ralph, E. Y. Tsymbal, M. S. Rzchowski, C. B. Eom, Controlling spin current polarization through non-collinear antiferromagnetism. *Nat. Commun.* **11**, 4671 (2020).

11. S. Nakatsuji, N. Kiyohara, T. Higo, Large anomalous Hall effect in a non-collinear antiferromagnet at room temperature. *Nature* **527**, 212–215 (2015).

12. N. Kiyohara, T. Tomita, S. Nakatsuji, Giant anomalous hall effect in the chiral antiferromagnet Mn_3Ge. *Phys. Rev. Appl.* **5**, 064009 (2016).

13. S. Novakov, P. B. Meisenheimer, G. A. Pan, P. Kezer, N. M. Vu, A. J. Grutter, R. F. Need, J. A. Mundy, J. T. Heron, Composite spin hall conductivity from non-collinear antiferromagnetic order. *Adv. Mater.* **35**, e2209866 (2023).

14. H. Chen, Q. Niu, A. H. Macdonald, Anomalous hall effect arising from noncollinear antiferromagnetism. *Phys. Rev. Lett.* **112**, 017205 (2014).

15. C. X. Quintela, K. Song, D. F. Shao, L. Xie, T. Nan, T. R. Paudel, N. Campbell, X. Pan, T. Tybell, M. S. Rzchowski, E. Y. Tsymbal, S. Y. Choi, C. B. Eom, Epitaxial antiperovskite/perovskite heterostructures for materials design. *Sci. Adv.* **6**, eaba4017 (2020).

16. B. H. Rimmler, B. K. Hazra, B. Pal, K. Mohseni, J. M. Taylor, A. Bedoya-Pinto, H. Deniz, M. Tangi, I. Kostanovskiy, C. Luo, R. R. Neumann, A. Ernst, F. Radu, I. Mertig, H. L. Meyerheim, S. S. P. Parkin, Atomic displacements enabling the observation of the anomalous hall effect in a non-collinear antiferromagnet. *Adv. Mater.* **35**, e2209616 (2023).

17. D. Boldrin, I. Samathrakis, J. Zemen, A. Mihai, B. Zou, F. Johnson, B. D. Esser, D. W. McComb, P. K. Petrov, H. Zhang, L. F. Cohen, Anomalous Hall effect in noncollinear antiferromagnetic Mn_3NiN thin films. *Phys. Rev. Mater.* **3**, 094409 (2019).

18. G. Gurung, D. F. Shao, T. R. Paudel, E. Y. Tsymbal, Anomalous Hall conductivity of noncollinear magnetic antiperovskites. *Phys. Rev. Mater.* **3**, 44409 (2019).

19. G. Gurung, D. F. Shao, E. Y. Tsymbal, Spin-torque switching of noncollinear antiferromagnetic antiperovskites. *Phys. Rev. B* **101**, 140405 (2020).

20. K. S. Ryu, L. Thomas, S. H. Yang, S. Parkin, Chiral spin torque at magnetic domain walls. *Nat. Nanotechnol.* **8**, 527–533 (2013).

21. T. Higo, H. Man, D. B. Gopman, L. Wu, T. Koretsune, O. M. J. van't Erve, Y. P. Kabanov, D. Rees, Y. Li, M.-T. Suzuki, S. Patankar, M. Ikhlas, C. L. Chien, R. Arita, R. D. Shull, J. Orenstein, S. Nakatsuji, Large magneto-optical Kerr effect and imaging of magnetic octupole domains in an antiferromagnetic metal. *Nat. Photonics* **12**, 73–78 (2018).

22. D. Fruchart, E. F. Bertaut, R. Madar, G. Lorthioir, R. Fruchart, Structure magnetique et rotation de spin de Mn_3NiN. *Solid State Commun.* **9**, 1793–1797 (1971).

23. I. Kounta, H. Reichlova, D. Kriegner, R. L. Seeger, A. Bad, M. Leiviska, A. Boussadi, V. Heresanu, S. Bertaina, M. Petit, E. Schmoranzerova, L. Smejkal, J. Sinova, T. Jungwirth, V. Baltz, S. T. B. Goennenwein, L. Michez, Competitive actions of MnSi in the epitaxial growth of Mn_5Si_3 thin films on Si (111). *Phys. Rev. Mater.* **7**, 024416 (2023).

24. F. Johnson, J. Kimák, J. Zemen, Z. Šobáň, E. Schmoranzerová, J. Godinho, P. Němec, S. Beckert, H. Reichlová, D. Boldrin, J. Wunderlich, L. F. Cohen, Identifying the octupole antiferromagnetic domain orientation in Mn_3NiN by scanning anomalous Nernst effect microscopy. *Appl. Phys. Lett.* **120**, 23 (2022).

25. B. H. Rimmler, B. Pal, S. S. P. Parkin, Non-collinear antiferromagnetic spintronics. *Nat. Rev. Mater.* **10**, 109–127 (2025).

26. H. Kawamura, Spin- and chirality-orderings of frustrated magnets- stacked-triangular anti-ferromagnets and spin glasses. *Can. J. Phys.* **79**, 1447–1458 (2001).

27. R. Takagi, R. Hirakida, Y. Settai, R. Oiwa, H. Takagi, A. Kitaori, K. Yamauchi, H. Inoue, J. I. Yamaura, D. Nishio-Hamane, S. Itoh, S. Aji, H. Saito, T. Nakajima, T. Nomoto, R. Arita, S. Seki, Spontaneous Hall effect induced by collinear antiferromagnetic order at room temperature. *Nat. Mater.* **24**, 63–68 (2025).

28. J. Xia, P. T. Beyersdorf, M. M. Fejer, A. Kapitulnik, Modified Sagnac interferometer for high-sensitivity magneto-optic measurements at cryogenic temperatures. *Appl. Phys. Lett.* **89**, 6 (2006).

29. S. F. Weber, A. Urru, S. Bhowal, C. Ederer, N. A. Spaldin, Surface magnetization in antiferromagnets: Classification, example materials, and relation to magnetoelectric responses. *Phys. Rev. X* **14**, 21033 (2024).

30. Y. Yang, Z. Luo, H. Wu, Y. Xu, R. W. Li, S. J. Pennycook, S. Zhang, Y. Wu, Anomalous Hall magnetoresistance in a ferromagnet. *Nat. Commun.* **9**, 2255 (2018).

31. Y. Tian, L. Ye, X. Jin, Proper scaling of the anomalous Hall effect. *Phys. Rev. Lett.* **103**, 087206 (2009).

32. D. Hou, Y. Li, D. Wei, D. Tian, L. Wu, X. Jin, The anomalous Hall effect in epitaxial face-centered-cubic cobalt films. *J. Phys. Condens. Matter* **24**, 482001 (2012).

33. I. M. L. Billas, A. Châtelain, W. A. de Heer, Magnetism from the atom to the bulk in iron, cobalt, and nickel clusters. *Science* **265**, 1682–1684 (1994).

34. L. Ye, Y. Tian, X. Jin, D. Xiao, Temperature dependence of the intrinsic anomalous Hall effect in nickel. *Phys. Rev. B* **85**, 220403 (2012).

35. K. Zhao, T. Hajiri, H. Chen, R. Miki, H. Asano, P. Gegenwart, Anomalous Hall effect in the noncollinear antiferromagnetic antiperovskite $Mn_3Ni_{1-x}Cu_xN$. *Phys. Rev. B* **100**, 045109 (2019).

36. D. Boldrin, F. Johnson, R. Thompson, A. P. Mihai, B. Zou, J. Zemen, J. Griffiths, P. Gubeljak, K. L. Ormandy, P. Manuel, D. D. Khalyavin, B. Ouladdiaf, N. Qureshi, P. Petrov, W. Branford, L. F. Cohen, The biaxial strain dependence of magnetic order in spin frustrated Mn_3NiN thin films. *Adv. Funct. Mater.* **29**, 1902502 (2019).

37. H. Reichlova, T. Janda, J. Godinho, A. Markou, D. Kriegner, R. Schlitz, J. Zelezny, Z. Soban, M. Bejarano, H. Schultheiss, P. Nemec, T. Jungwirth, C. Felser, J. Wunderlich, S. T. B. Goennenwein, Imaging and writing magnetic domains in the non-collinear antiferromagnet Mn3Sn. *Nat. Commun.* **10**, 5459 (2019).

38. S. Li, M. Huang, H. Lu, N. J. McLaughlin, Y. Xiao, J. Zhou, E. E. Fullerton, H. Chen, H. Wang, C. R. Du, Nanoscale magnetic domains in polycrystalline Mn_3Sn films imaged by a scanning single-spin magnetometer. *Nano Lett.* **23**, 5326–5333 (2023).

39. S. Thomas, B. Kuiper, J. Hu, J. Smit, Z. Liao, Z. Zhong, G. Rijnders, A. Vailionis, R. Wu, G. Koster, J. Xia, Localized control of curie temperature in perovskite oxide film by capping-layer-induced octahedral distortion. *Phys. Rev. Lett.* **119**, 177203 (2017).

40. C. Gong, L. Li, Z. Li, H. Ji, A. Stern, Y. Xia, T. Cao, W. Bao, C. Wang, Y. Wang, Z. Q. Qiu, R. J. Cava, S. G. Louie, J. Xia, X. Zhang, Discovery of intrinsic ferromagnetism in two-dimensional van der Waals crystals. *Nature* **546**, 265–269 (2017).

41. C. Farhang, W. Lu, Y. Yao, P. Pal, S. Han, J.-G. Zheng, H. Chen, C.-B. Eom, J. Xia, Temperature-invariant magneto-optical Kerr effect in a noncollinear antiferromagnet. arXiv: 2510.19709 [cond-mat.str-el] (2025).

42. H. B. Callen, E. Callen, The present status of the temperature dependence of magnetocrystalline anisotropy, and the l(l+1) 2 power law. *J. Phys. Chem. Solid* **27**, 1271–1285 (1966).

43. P. Asselin, R. F. L. Evans, J. Barker, R. W. Chantrell, R. Yanes, O. Chubykalo-Fesenko, D. Hinzke, U. Nowak, Constrained Monte Carlo method and calculation of the temperature dependence of magnetic anisotropy. *Phys. Rev. B* **82**, 054415 (2010).

44. R. F. L. Evans, U. Atxitia, R. W. Chantrell, Quantitative simulation of temperature-dependent magnetization dynamics and equilibrium properties of elemental ferromagnets. *Phys. Rev. B* **91**, 144425 (2015).

45. David. Vanderbilt, Soft self-consistent pseudopotentials in a generalized eigenvalue formalism. *Phys. Rev. B* **41**, 7892–7895 (1990).

46. P. Giannozzi, S. Baroni, N. Bonini, M. Calandra, R. Car, C. Cavazzoni, D. Ceresoli, G. L. Chiarotti, M. Cococcioni, I. Dabo, A. Dal Corso, S. De Gironcoli, S. Fabris, G. Fratesi, R. Gebauer, U. Gerstmann, C. Gougoussis, A. Kokalj, M. Lazzeri, L. Martin-Samos, N. Marzari, F. Mauri, R. Mazzarello, S. Paolini, A. Pasquarello, L. Paulatto, C. Sbraccia, S. Scandolo, G. Sclauzero, A. P. Seitsonen, A. Smogunov, P. Umari, R. M. Wentzcovitch, QUANTUM ESPRESSO: A modular and open-source software project for quantum simulations of materials. *J. Phys. Condens. Matter* **21**, 395502 (2009).

47. J. P. Perdew, K. Burke, M. Ernzerhof, Generalized gradient approximation made simple. *Phys. Rev. Lett.* **77**, 3865–3868 (1996).

48. M. B. Nardelli, F. T. Cerasoli, M. Costa, S. Curtarolo, R. De Gennaro, M. Fornari, L. Liyanage, A. R. Supka, H. Wang, PAOFLOW: A utility to construct and operate on ab initio Hamiltonians from the projections of electronic wavefunctions on atomic orbital bases, including characterization of topological materials. *Comput. Mater. Sci.* **143**, 462–472 (2018).

49. L. A. Agapito, A. Ferretti, A. Calzolari, S. Curtarolo, M. Buongiorno Nardelli, Effective and accurate representation of extended Bloch states on finite Hilbert spaces. *Phys. Rev. B* **88**, 165127 (2013).

50. L. A. Agapito, S. Ismail-Beigi, S. Curtarolo, M. Fornari, M. B. Nardelli, Accurate tight-binding Hamiltonian matrices from ab initio calculations: Minimal basis sets. *Phys. Rev. B* **93**, 035104 (2016).

51. R. F. L. Evans, W. J. Fan, P. Chureemart, T. A. Ostler, M. O. A. Ellis, R. W. Chantrell, Atomistic spin model simulations of magnetic nanomaterials. *J. Phys. Condens. Matter* **26**, 103202 (2014).

52. J. D. Alzate-Cardona, D. Sabogal-Suárez, R. F. L. Evans, E. Restrepo-Parra, Optimal phase space sampling for Monte Carlo simulations of Heisenberg spin systems. *J. Phys. Condens. Matter* **31**, 095802 (2019).

53. W. Feng, G. Y. Guo, J. Zhou, Y. Yao, Q. Niu, Large magneto-optical Kerr effect in noncollinear antiferromagnets Mn_3X (X=Rh, Ir, Pt). *Phys. Rev. B* **92**, 144426 (2015).

54. Z. Q. Liu, H. Chen, J. M. Wang, J. H. Liu, K. Wang, Z. X. Feng, H. Yan, X. R. Wang, C. B. Jiang, J. M. D. Coey, A. H. Macdonald, Electrical switching of the topological anomalous hall effect in a non-collinear antiferromagnet above room temperature. *Nat. Electron.* **1**, 172–177 (2018).
